# Natural small molecules regulating the mitophagy pathway counteract the pathogenesis of diabetes and chronic complications

**DOI:** 10.3389/fphar.2025.1571767

**Published:** 2025-04-16

**Authors:** Du Ye, Junping Zhu, Siya Su, Yunfeng Yu, Jun Zhang, Yuman Yin, Chuanquan Lin, Xuejiao Xie, Qin Xiang, Rong Yu

**Affiliations:** ^1^ College of Chinese Medicine, Hunan University of Chinese Medicine, Changsha, Hunan, China; ^2^ The Second Hospital of Hunan University of Chinese Medicine, Changsha, Hunan, China; ^3^ School of Informatics, Hunan University of Chinese Medicine, Changsha, Hunan, China; ^4^ Guangzhou University of Chinese Medicine, Guangzhou, Guangdong, China

**Keywords:** mitophagy, natural small molecules, diabetes mellitus, signaling pathway, review

## Abstract

Diabetes mellitus (DM) is a chronic metabolic disorder marked by sustained hyperglycemia. These disturbances contribute to extensive damage across various tissues and organs, giving rise to severe complications such as vision loss, kidney failure, amputations, and higher morbidity and mortality rates. Furthermore, DM imposes a substantial economic and emotional burden on patients, families, and healthcare systems. Mitophagy, a selective process that targets the clearance of damaged or dysfunctional mitochondria, is pivotal for sustaining cellular homeostasis through mitochondrial turnover and recycling. Emerging evidence indicates that dysfunctional mitophagy acts as a key pathogenic driver in the pathogenesis of DM and its associated complications. Natural small molecules are particularly attractive in this regard, offering advantages such as low toxicity, favorable pharmacokinetic profiles, excellent biocompatibility, and a broad range of biochemical activities. This review systematically evaluates the mechanistic roles of natural small molecules—including ginsenosides, resveratrol, and berberine—in enhancing mitophagy and restoring mitochondrial homeostasis via activation of core signaling pathways (e.g., PINK1/Parkin, BNIP3/NIX, and FUNDC1). These pathways collectively ameliorate pathological hallmarks of DM, such as oxidative stress, chronic inflammation, and insulin resistance. Furthermore, the integration of nanotechnology with these compounds optimizes their bioavailability and tissue-specific targeting, thereby establishing a transformative therapeutic platform for DM management. Current evidence demonstrates that mitophagy modulation by natural small molecules not only offers novel therapeutic strategies for DM and its chronic complications but also advances the mechanistic foundation for future drug development targeting metabolic disorders.

## 1 Introduction

Diabetes mellitus (DM) is a chronic condition featured with persistently elevated blood glucose levels, which disrupt normal metabolic processes and lead to various systemic complications (Yu et al., 2024). In the light of the Global Burden of Disease study published in *The Lancet*, an estimated 828 million adults (aged 18 years and older) globally were living with DM in 2022, with an age-standardized prevalence rate of 13.9% in women and 14.3% in men (Zhou et al., 2024). Data indicate that the global DM epidemic is worsening, with increasing prevalence in younger populations over time (Stene, 2024). DM and its complications impose significant financial and psychological burdens on individuals, families, and society (The Lancet Diabetes and Endocrinology, 2024). Hyperglycemia, along with long-term metabolic disturbances, exacerbates disease progression, leading to widespread tissue and organ damage and contributing to severe complications. Despite the availability of various preventive and therapeutic interventions, the complex and multifactorial nature of DM makes effective treatment challenging. Thus, a deeper understanding of its pathogenesis is crucial for developing targeted therapeutic strategies to manage DM and its complications. Recent research has underscored the critical involvement of mitophagy in the pathophysiology (Ajoolabady et al., 2022; Ke, 2020; Wang et al., 2023a). Mitophagy, a selective process that targets the clearance of damaged or dysfunctional mitochondria, is pivotal for sustaining cellular homeostasis through mitochondrial turnover and recycling (Picca et al., 2023). Emerging evidence suggests that the proper modulation of mitophagy is critical in ensuring metabolic balance in DM, and its dysfunction may be a contributing factor to the onset and progression of associated complications (Yang et al., 2024a; Zhu et al., 2024; Sriwastawa and Kumar, 2024), positioning it as a promising biomarker and potential therapeutic target. Natural small molecules, a class of bioactive compounds with low molecular weight, are derived from plants, animals, microorganisms, or synthetic methods. These molecules are known for their low toxicity, high druggability, excellent biocompatibility, and diverse biochemical activities (Clardy and Walsh, 2004; Ortholand and Ganesan, 2004). They have demonstrated considerable effectiveness in preventing and mitigating the complications of DM. For example, natural small molecules such as ginsenosides and resveratrol have been shown to ameliorate pathological hallmarks of diabetes (e.g., oxidative stress and insulin resistance) by modulating mitophagy pathways (Li et al., 2023a; Wang et al., 2018a). This review offers a comprehensive analysis of the significance of mitophagy and its related pathways in DM and its complications. It highlights the current advancements in the study of natural small molecules that orchestrate mitophagy to address DM and its associated complications. The findings presented in this review lay a solid groundwork for future investigations and the formulation of innovative therapeutic approaches aimed at combating mitochondrial dysfunction in metabolic disorders.

## 2 Regulatory pathways of mitophagy

**FIGURE 1:**
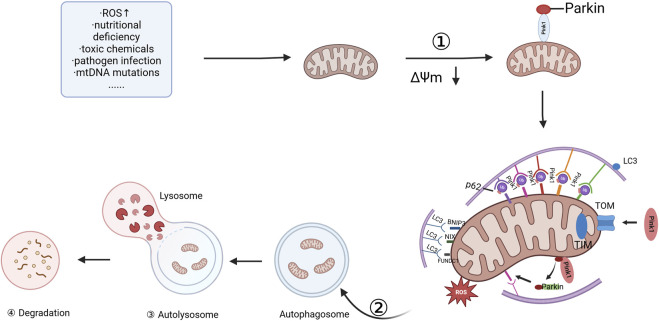
Committed step of mitophagy. ① depolarization of damaged mitochondria; ② wrapping of mitochondria by autophagosomes; ③ fusion of mitochondrial autophagosomes with lysosomes; and ④ mitochondrial contents are degraded by lysosomes, and lysosomal or vesicular acid hydrolases flow into autophagosomes to degrade damaged mitochondria. The figure is drawn with biorender.com.

Mitophagy, or mitophagy, was first defined by Lemasters in 2005 (Lemasters, 2005) as a highly regulated process that selectively eliminates damaged or dysfunctional mitochondria to maintain cellular homeostasis. This process involves a series of well-coordinated steps, each crucial for the efficient clearance of defective mitochondria (Palikaras et al., 2018). Mitophagy involves four key steps: first, damaged mitochondria undergo depolarization, leading to a loss of mitochondrial membrane potential (MMP). Second, the damaged mitochondria are then enveloped by autophagosomes, forming mitochondrial autophagosomes. Third, the mitochondrial autophagosomes subsequently fuse with lysosomes, where their contents are degraded. Finally, the lysosome degrades the mitochondrial components, completing the process of mitochondrial quality control (MQC) (Lyamzaev et al., 2018; Samuvel et al., 2022; Miyamoto et al., 2011). Mitophagy serves as a quality control mechanism, ensuring that only healthy, functional mitochondria remain in the cellular network. By selectively eliminating damaged mitochondria, it helps maintain mitochondrial dynamics and cellular function. A summary of these key steps is depicted in Figure 1.

Mitophagy, a process essential for cellular homeostasis, can be classified into two main pathways: the ubiquitin- and the non-ubiquitin-dependent pathways (Palikaras et al., 2018; Khaminets et al., 2016).

The ubiquitin-dependent pathway involves the tagging of mitochondrial surface proteins with ubiquitin, facilitating their recognition and subsequent degradation (Fritsch et al., 2020). This pathway encompasses both the well-known PINK1-Parkin-mediated mitophagy and alternative, Parkin-independent mechanisms. The PINK1/Parkin axis is the most extensively researched and thoroughly understood mechanism responsible for the targeted removal of dysfunctional mitochondria in mammalian cells (Imberechts et al., 2022; Liu et al., 2023a; Li et al., 2023b). PINK1 is a highly conserved mitochondrial protein that contains a kinase domain and mitochondrial localization sequences, while Parkin is an E3 ubiquitin ligase found in the cytosol. Parkin is responsible for recognizing and ubiquitinating target proteins on damaged mitochondria (Han et al., 2023). The sequence of events in PINK1/Parkin-mediated mitophagy begins when mitochondria become damaged. A loss of MMP prevents PINK1 from entering the inner mitochondrial membrane, causing it to accumulate on the outer membrane. This accumulation acts as a signal, recruiting Parkin from the cytosol to the damaged mitochondria. Upon recruitment, Parkin undergoes a conformational change, activating Its E3 ligase activity. This activation results in the ubiquitination of outer mitochondrial membrane proteins, leading to the formation of protein aggregates that are then recognized by the autophagic receptor protein P62 (Riley et al., 2013). The P62 protein interacts with LC3, facilitating the production of autophagic lysosomes that degrade the damaged mitochondria. This process is highly coordinated, with PINK1 and Parkin working together to orchestrate MQC and ensure mitochondrial homeostasis (Whitworth and Pallanck, 2009). In addition to the canonical PINK1/Parkin axis, there is increasing evidence of a Parkin-independent, ubiquitin-dependent pathway. This pathway implicates other E3 ligases such as MUL1, Gp78, SMURF1, ARIH1, SIAH1, and RNF185 (Igarashi et al., 2020; Liu et al., 2012; Zhang et al., 2022a; Mukherjee and Chakrabarti, 2016). These autophagy receptors bind to the ubiquitinated proteins and initiate mitophagy in a Parkin-independent fashion (Lu et al., 2023).

The non-ubiquitin-dependent pathway involves several receptor proteins that mediate mitophagy and are associated with mitochondrial dynamics. These receptors are classified into two groups: one group contains ubiquitin-binding domains, such as p62, OPTN, and NDP52, while the other group lacks these domains, including C-cbl, NCOA4, BNIP3L/NIX, FUNDC1, STBD1, PHB2, etc (Liu et al., 2012; Xie et al., 2020; Bellot et al., 2009). These receptors facilitate mitophagy by directly binding to LC3, without the need for ubiquitination. Mitochondrial dynamics is critical in ensuring mitochondrial mass through fission and fusion processes. When this balance is disrupted, damaged mitochondria are selectively removed via mitophagy, while healthy mitochondria are reintegrated into the network through fusion. This process, referred to as mitochondrial dynamics-mediated mitophagy, is critical for ensuring cellular energy supply and function (Shirihai et al., 2015; Lacombe and Scorrano, 2024; Xiao et al., 2022). Several pathways are involved in regulating mitophagy. For example, the FOXO1/PINK1 pathway enhances the expression of mitophagy-related proteins such as LC3II, PINK1, and Parkin, while reducing the expression of senescence-related genes (e.g., Rb, p16, p21). This pathway improves insulin sensitivity, fasting insulin levels, and fasting glucose levels in DM mice. As a mitochondrial outer membrane receptor, FUNDC1 orchestrates hypoxia-induced mitophagy through LC3 interaction via its LIR (LC3-interacting region) domain, serving as a critical regulatory node in stress-responsive mitochondrial quality control (Liu et al., 2012). The FUNDC1 pathway is activated under hypoxic conditions, where FUNDC1 binds to LC3, promoting the association of mitochondria with autophagosomes and accelerating mitophagy (Dong et al., 2022). Additionally, the serine/threonine kinase ULK1 phosphorylates FUNDC1, which is critical for the targeting of FUNDC1 to damaged mitochondria during mitophagy (Zhu et al., 2022). FUNDC1, a hypoxia-inducible mitochondrial outer membrane receptor, mediates stress-responsive mitophagy by recruiting ULK1 to phosphorylate Ser17, thereby initiating autophagosome formation under conditions of oxygen deprivation or mitochondrial membrane depolarization (Wu et al., 2014). Concurrently, the AMPK-mTORC1 axis serves as a central energy-sensing regulator of this process: under nutrient-replete conditions, mTORC1 phosphorylates ULK1 at Ser757 to suppress mitophagy by disrupting its interaction with AMPK (Saxton and Sabatini, 2017); conversely, energy depletion (e.g., hypoxia-induced ATP decline) activates AMPK through elevated AMP/ATP ratios, which concurrently phosphorylates ULK1 at Ser317/Ser777 to trigger autophagosome assembly and inactivates mTORC1 via TSC2-Rheb signaling, thereby relieving ULK1 inhibition (Dengler, 2020; Kim et al., 2013; Inoki et al., 2003). Complementing these pathways, the NAD ± dependent deacetylase SIRT1 enhances mitophagy through dual mechanisms—direct deacetylation of LC3 to facilitate autophagosomal membrane expansion (Huang et al., 2015) and activation of PGC1α, a transcriptional coactivator that upregulates mitochondrial biogenesis genes (e.g., TFAM, NRF1) while synergistically promoting mitochondrial turnover (Hwang and Song, 2017; Cantó et al., 2010; Jäger et al., 2007). Nicotinamide (NAM) amplifies this cascade by stabilizing cellular NAD + pools, thereby potentiating SIRT1 activity and linking metabolic sensing to mitochondrial quality control (Hwang and Song, 2017). The BNIP3 and BNIP3L/NIX pathways are also critical in mitochondrial clearance. These proteins interact with LC3 to promote the removal of dysfunctional mitochondria and maintain mitochondrial homeostasis. Their function is modulated by the formation of homo- and heterodimers, which are necessary for ensuring mitochondrial integrity (Hendgen-Cotta et al., 2017). BNIP3, a pro-apoptotic Bcl-2 family member sharing BH3 domain homology, functions as a hypoxia-inducible mitophagy receptor. Under hypoxic stress, BNIP3 undergoes transcriptional upregulation and translocates to the mitochondrial outer membrane via its C-terminal transmembrane domain. Structural stabilization occurs through homodimerization, while its N-terminal LC3-interacting region (LIR) motifs engage LC3 through phosphorylation-dependent mechanisms at Ser^17^ and Ser^24^ residues to drive mitophagosome formation (Hanna et al., 2012; Zhu et al., 2013). NIX (BNIP3L), a mitochondrial outer membrane protein exhibiting 56% sequence homology with BNIP3, orchestrates selective mitochondrial degradation during reticulocyte maturation. This process requires phosphorylation at Ser^34^/Ser^35^ to enhance LIR-mediated binding to LC3A/B isoforms, coupled with redox-sensitive ROS accumulation and Rheb GTPase signaling (Rogov et al., 2017; Marinković et al., 2021). Notably, BNIP3 cross-talks with mTORC1 by sequestering Rheb, thereby alleviating mTORC1-mediated suppression of ULK1 kinase activity to potentiate mitophagy (Li et al., 2007; Melser et al., 2013). Both receptors interface with the PINK1/Parkin pathway: NIX undergoes Parkin-dependent ubiquitination to amplify damaged mitochondrial recognition, while BNIP3 facilitates PINK1 stabilization, promoting Parkin recruitment to depolarized mitochondria for proteasomal targeting (Gao et al., 2015; Zhang et al., 2016). Furthermore, BNIP3 and BNIP3L/NIX interact with mitochondrial phagocytosis protein Mieap and CDH6, facilitating the elimination of reactive oxygen species (ROS) and modulating Drp1-mediated mitochondrial fission (Nakamura et al., 2012). Recent studies have also identified new therapeutic targets, such as hexokinase-2, small ribosomal subunit protein US3, and L-lactate dehydrogenase A, which have demonstrated crucial roles in mitophagy in diabetic ulcers (Guo et al., 2024).

In summary, the regulation of mitophagy is a complex process governed by a network of physiological mechanisms, encompassing both ubiquitin- and non-ubiquitin-dependent pathways. These intricate pathways are vital in preserving MQC and ensuring optimal cellular functioning.

## 3 The role of mitophagy in DM and its chronic complications

**FIGURE 2:**
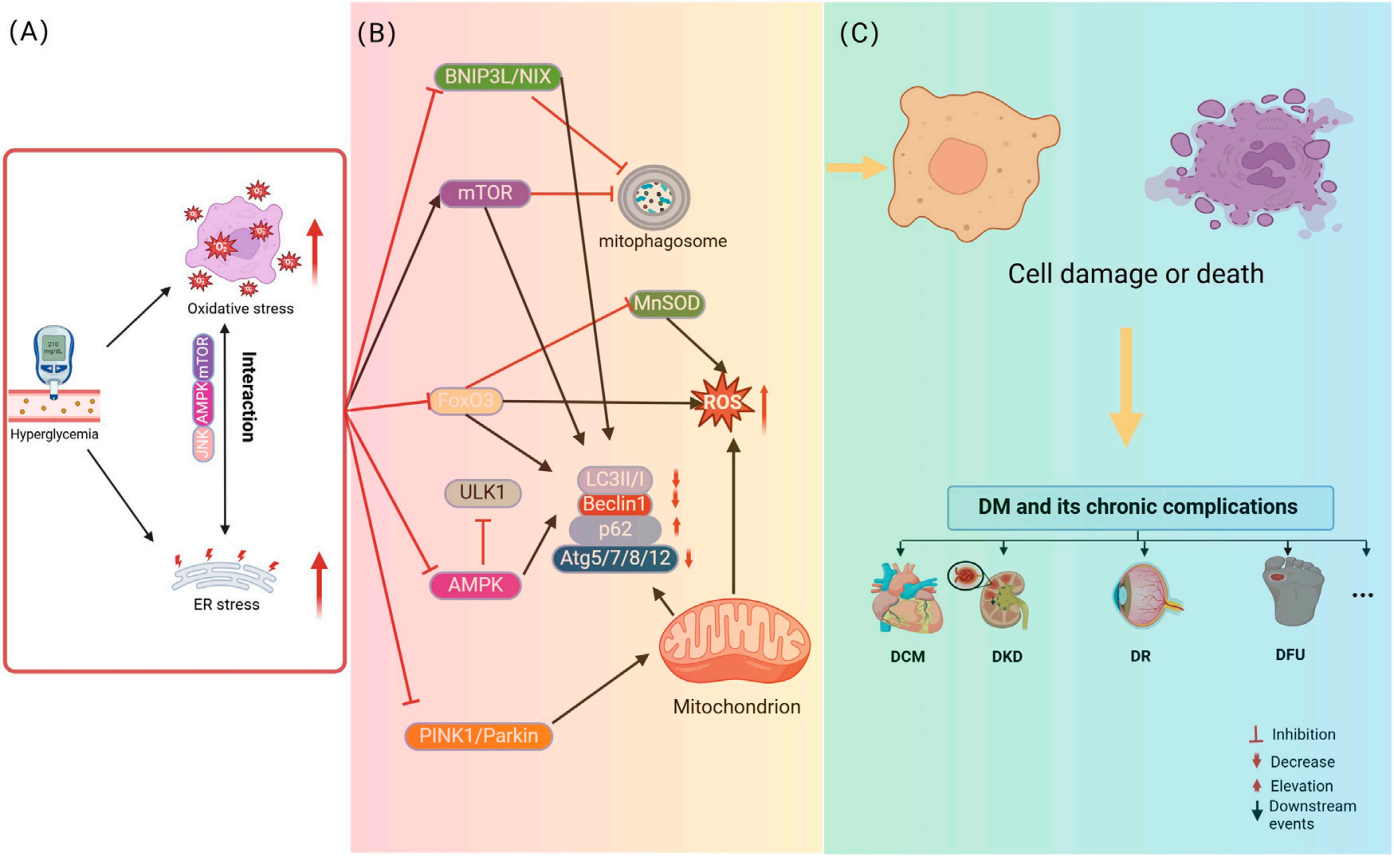
Hyperglycemia-driven mitophagy impairment in diabetic pathogenesis and complications: Synergistic roles of oxidative and endoplasmic reticulum stress. **(A)** describes that hyperglycemia initiates the process of cellular damage by inducing oxidative stress and ER stress, where oxidative stress and ER stress interact with each other through signaling pathways, such as JNK, to form a vicious cycle. **(B)** Revealed that hyperglycemia, oxidative stress and ER stress further lead to the disruption of mitochondria-related pathways, including mitophagy dysfunction. **(C)** Elucidated the consequences of mitophagy dysfunction, i.e., damaged mitochondria cannot be effectively removed, leading to cell damage or death, which ultimately contributes to the development of diabetes and its chronic complications. The figure is drawn with biorender.com.

Mitophagy dysfunction has been linked to the pathogenesis of numerous clinical conditions, with both excessive and insufficient autophagy identified as key factors in the onset and progression of various diseases. In the context of DM, recent research has highlighted the significance of mitophagy, suggesting that its regulation could offer potential therapeutic benefits. Specifically, moderate mitophagy is capable of alleviating symptoms of DM and its complications, while either underactive or overly active autophagy aggravates disease. Hyperglycemia-driven mitophagy impairment in diabetic pathogenesis and complications: Synergistic roles of oxidative and endoplasmic reticulum stress are illustrated in Figure 2.

Type 1 diabetes mellitus (T1DM) is a T cell-mediated autoimmune disease, and the pathogenesis is closely linked to mitophagy and mitochondrial function (Blagov et al., 2023). During the onset of T1DM, the immune system attacks pancreatic β-cells, and a variety of immune cells infiltrate, releasing pro-inflammatory cytokines, which triggers pancreatic islet inflammation, and promotes apoptosis of β-cells (Christianson et al., 1993). Abnormal mitochondrial function is also critical. Damaged mitochondria release mtROS and mtDNA. mtROS cause macrophages to activate and secrete pro-inflammatory factors, and mtDNA activates NLRP3 inflammasome, which drives inflammation and damage β cells (Carlos et al., 2017). Mitophagy is significant in T1DM. It maintains mitochondrial homeostasis in normal conditions, but its function is impaired in T1DM, resulting in the accumulation of damaged mitochondria, which activate inflammatory signaling pathways and trigger chronic inflammation and autoimmune responses (Mills et al., 2017). Compounds such as PMI and Urolithins can induce mitophagy, bringing new possibilities for the treatment of T1DM (East et al., 2014).

Gestational diabetes mellitus (GDM)is a diabetes mellitus that occurs during pregnancy and is closely related to insulin resistance and β-cell dysfunction. It has been shown that GDM leads to mitochondrial dysfunction in placental endothelial and trophoblast cells, especially reduced mitochondrial F0F1-ATP synthase (complex V) activity, which reduces placental oxidative phosphorylation capacity. This phenomenon may be related to the overproduction of superoxide anion and nitric oxide. In addition, the synergistic action of placental mitochondrial biosynthesis and mitophagy is essential for the maintenance of cellular homeostasis, especially during pregnancy, to ensure the efficient production of ATP required for fetal growth and development (Sobrevia et al., 2020).

In DM, defects in pancreatic β-cells or the development of insulin resistance (IR) impair insulin secretion, which ultimately results in persistent hyperglycemia (Jia et al., 2021). Previous studies have documented (Park et al., 2023; Čater and Križančić Bombek, 2022) a close link between mitochondrial dysfunction and IR. Damaged mitochondria accumulate within cells, and increased mitochondrial ROS production further exacerbates oxidative damage and glucose intolerance in β-cells. This oxidative stress (OS) contributes to β-cell apoptosis, worsening IR and creating a cycle that accelerates disease progression. Mitochondria also modulate insulin secretion via the modulation of ATP synthase activity and ATP production, both of which are crucial for normal β-cell function. Growing evidence points to mitophagy as a key regulator of mitochondrial health under hyperglycemic conditions, potentially playing a critical role in mitigating oxidative damage by removing dysfunctional mitochondria and excess ROS (Shan et al., 2022; Shim et al., 2023). For example, Kaniuka et al. (2025) demonstrated that glucotoxicity-induced elevation of GRP78 led to depletion of antioxidant reserves and impaired antioxidant defense mechanisms, which in turn promoted mitophagy in pancreatic cells, helping to mitigate the effects of DM in DM rats. Similarly, Oh et al. (2023) showed that mice with β-cell-specific deletion of Tfeb (Tfeb^Δ^ ^β-cell^) exhibited β-cell dysfunction and glucose intolerance following exposure to a calmodulin phosphatase inhibitor, which hindered TFEB activation and mitophagy. These findings further pinpoint the critical role of mitophagy in β-cell function. Overall, it is evident that mitochondrial dynamics and autophagy are impaired in DM, leading to dysregulated mitochondrial function and increased IR. Restoring proper mitophagy and dynamics could, therefore, offer a promising therapeutic avenue for addressing DM and its associated complications.

### 3.1 Microangiopathy

#### 3.1.1 Diabetic kidney disease

DKD is a significant complication of DM and a primary contributor to end-stage renal disease (Ahmad et al., 2021). Hyperglycemia-induced mitochondrial dysfunction has been documented as a key factor driving renal tubular epithelial cell (EC) damage in DKD. Liu et al. (2024) demonstrated that renal tubulointerstitial inflammation serves as a critical predictor for the progression of DKD. Their study revealed a marked increase in TRPC6 levels in DKD, which is pivotal in inhibiting mitophagy through the activation of calcineurin-1. Silencing Trpc6 helped partially restore mitophagy, thereby reducing both renal tubular injury (RTI) and tubulointerstitial inflammation in experimental models. In a separate study, Ji et al. (2024) showed that overexpression of UHRF1 promoted PINK1-mediated mitophagy by diminishing TXNIP expression, which in turn suppressed ferroptosis. These findings uncover that elevated UHRF1 can positively influence mitophagy and help alleviate DKD. Xiao et al. (2017) reported that Nrf2-mediated transcriptional regulation of PINK1 restored mitophagy in renal tubular cells, reducing mitochondrial OS and restoring mitochondrial dynamics. This ultimately alleviated RTI. Furthermore, Yang et al. (2024) reported that WJ-39 treatment in rats with DKD preserved mitochondrial integrity in renal tubules by inhibiting aldose reductase, activating the PINK1/Parkin axis, and promoting mitophagy. This intervention effectively alleviated mitochondrial apoptosis. These findings unveil the significance of mitophagy in DKD. Mitigating RTI by reducing mitochondrial OS in hyperglycemic conditions presents a promising strategy for the therapeutic intervention in DKD.

#### 3.1.2 Diabetic retinopathy

DR is a severe complication of DM and remains the leading cause of blindness on a global scale (Sun et al., 2022). Numerous studies have suggested (Liu et al., 2023b; Wang et al., 2023b) that mitochondrial dysfunction is critical in OS, inflammation, and apoptosis observed in the retina of DR patients. Mitophagy has emerged as a key area of study, as it appears to play a dual role in DR, with both beneficial and deleterious effects. Zhang et al. (2022b) found that TGR5 ameliorated vascular endothelial cell (VEC) dysfunction in DR by inhibiting mitochondrial fission through the regulation of the PKCδ/Drp1-HK2 axis, while also enhancing mitophagy in RMECs. Similarly, Yang et al. (2024c) reported that overexpression of Sirt3 elevated p-AMPK/AMPK and p-ULK1/ULK1, while decreasing p-mTOR/mTOR expression. This regulatory effect inhibited apoptosis and promoted mitophagy, which protected RPE cells from high-glucose (HG)-induced injury. Furthermore, Serikbaeva et al. (2022) found that HG levels promoted mitophagy, which in turn helped attenuate HG-induced injury in DR. Additionally, Zhang et al. (2024) observed that knockdown of TIN2 distinctly declined cellular senescence and mitochondrial OS in ARPE-19 cells under HG conditions. This intervention restored retinal thickness and RPE cell tight junctions in DR mice. The increased mitochondrial localization of TIN2 induced cellular senescence in RPE cells by impairing mitophagy and activating mTOR signaling. The harmful effects of mitophagy in DR were further explored by Cai et al. (2017), who investigated OS-induced apoptosis and autophagy in the retinas of T2DM rats induced by a HG, high-fat diet (HFD) and a single injection of streptozotocin (STZ). Their findings showed increased levels of LC3-II/I, along with enhanced OS enzyme activities such as reductive nicotinamide adenine dinucleotide phosphoryl oxidase 3 and superoxide dismutase 2. The increased autophagy activity inhibited apoptosis and OS, providing evidence that modulating autophagy levels could mitigate the progression of DR. Additional studies support the complex role of mitophagy in DR pathogenesis. For instance, Wu et al. (2022a) demonstrated that knockdown of Drp1 inhibited retinal endothelial cell apoptosis in rats by suppressing mitophagy. Li et al. (2019a) showed that inducing mitophagy promoted tube formation and migration of choroid-retinal endothelial cells in monkeys, suggesting a role for mitophagy in enhancing angiogenesis. In summary, mitophagy plays a complex, dual role in DR.

#### 3.1.3 Diabetic cardiomyopathy

DCM is a severe complication of DM, characterized by systolic dysfunction and left ventricular hypertrophy, leading to significant impairment in cardiac performance (Lu et al., 2021). Mitophagy is recognized as a protective mechanism in DCM, as it helps to eliminate damaged mitochondria, alleviates OS, and reduces cardiomyocyte apoptosis (Bai et al., 2020). A study by Mu et al. (2020) explored the role of BRD4 in DCM, showing that elevation of BRD4 in the hearts of DM mice repressed PINK1/Parkin-mediated mitophagy. This inhibition resulted in the generation of damaged mitochondria, which, in turn, led to structural and functional deterioration of the heart. In contrast, the use of JQ1, a BRD4 inhibitor, was found to enhance mitochondrial function and restore the structural and functional integrity of the diabetic heart. Notably, Pink1 deficiency was found to exacerbate DCM by inhibiting mitophagy, highlighting the importance of mitophagy in mitigating myocardial damage. These findings suggest that reduced mitophagy in the diabetic myocardium contributes to myocardial injury, and restoring mitophagy could help alleviate cardiomyocyte damage in DM-related heart disease.

### 3.2 Atherosclerotic cardiovascular disease

Atherosclerosis is notably prevalent among individuals with DM and tends to progress at an accelerated rate, significantly heightening the risk of cardiovascular and cerebrovascular diseases, which remain leading causes of mortality in DM patients. Atherosclerosis primarily impacts major arteries such as the aortic, coronary, cerebral, renal, and limb arteries, contributing to conditions such as coronary artery disease, ischemic or hemorrhagic stroke, renal arteriosclerosis, and peripheral artery disease. A key mechanism driving the progression of ASCVD is endothelial cell injury, with hyperglycemia playing a critical role in inducing endothelial dysfunction. Mitophagy has emerged as a protective mechanism against hyperglycemia-induced damage in endothelial cells. A previous study (Zhu et al., 2018) demonstrated that PINK1/Parkin-mediated mitophagy alleviated mitochondrial dysfunction in DM rats, thus protecting endothelial cells from hyperglycemia-induced damage. Similarly, Liu et al. (2017a) found that exogenous hydrogen sulfide (H_2_S) protected rat aortic endothelial cells (RAECs) from HG and palmitate-induced injury by repressing OS, reducing mitochondrial fragmentation, and promoting mitophagy. This intervention helped prevent RAEC apoptosis under HG and palmitate conditions. These results suggest that impaired mitophagy in diabetic endothelial cells exacerbates cellular damage. Therefore, restoring mitophagy in these cells may provide a novel therapeutic approach to mitigate endothelial cell injury in DM and reduce the risk of ASCVDs.

### 3.3 Neurological complications

#### 3.3.1 Central nervous system complications

CNS complications in DM include ➀ altered mental status, typically associated with severe diabetic ketoacidosis (DKA), hyperosmolar hyperglycemic syndrome, or hypoglycemia; ➁ stroke; and ➂ accelerated brain aging, including Alzheimer’s disease (AD). Mitophagy is crucial in mitigating CNS complications associated with AD and T2DM (Paul et al., 2021). Mitophagy is considered one of the primary mechanisms that alleviates CNS dysfunction in these conditions. For instance, in DM-related stroke, neurodegeneration may occur due to severe ischemia and hypoxia in the ischemic core of the brain (Szydlowska and Tymianski, 2010). Reestablishment of cerebral blood flow after acute stroke treatment, although essential, can lead to ischemia/reperfusion (I/R) injury, which exacerbates neuronal damage (Leech et al., 2019). Excessive mitochondrial dysfunction due to I/R injury is a major contributor to neuronal apoptosis, which is a key cause of cell death in stroke (Wang et al., 2022a; Nhu et al., 2021). As documented by previous studies, mitophagy is activated following brain I/R injury, helping to mitigate this damage (Lan et al., 2018; Cai et al., 2021). For example, Yaqi Guo et al. demonstrated that metformin alleviated hyperglycemia-exacerbated brain I/R injury by activating the AMPK/ULK1/PINK1/Parkin mitophagy axis (Guo et al., 2023).

#### 3.3.2 Diabetic peripheral neuropathy

DPN is a prevalent complication of chronic DM, resulting from impaired blood flow to peripheral nerves, which leads to neuronal dysfunction. This dysfunction manifests through deficits in nerve conduction and other neuropathic symptoms (Khan et al., 2023). Mitophagy plays a significant role in ameliorating DPN. Zheng et al. (2024) found that hyperbaric lidocaine (HL) induced PINK1-mediated mitophagy via the activation of the p38 MAPK/ERK axis, which exacerbated spinal cord tissue injury in DNP rats. Yang et al. (2024d) demonstrated that overexpression of SIRT3 induced mitophagy via activation of the FoxO3a-PINK1-Parkin axis, thereby improving DPN. Furthermore, Yuan et al. (2022a) identified that defects in mitophagy, induced by PARP1 in dorsal root ganglion neurons, were a critical mechanism underlying DM-induced peripheral nerve damage. In a related study, Khan et al. (2024) validated that silibinin elevated the expression of PARL, PINK1, PGAM5, and LC3, ensuring mitophagy through various mechanisms. Additionally, silibinin promoted antioxidant defense by upregulating Nrf2, and enhanced the expression of SIRT1, PGC-1α, and TFAM in sciatic nerves and HG-injured N2A cells. Collectively, these findings pinpoint the significance of mitophagy in the pathogenesis of DPN, suggesting that targeted modulation of mitochondrial autophagic processes could offer a promising therapeutic modality for treating this condition.

### 3.4 Diabetic foot ulcer

DFU is a severe complication in DM, with many patients at risk of amputation (Jeffcoate et al., 2018). DFUs primarily result from the interplay of multiple factors, including neuropathy, impaired vascularization, and biomechanical abnormalities. The incidence of DFU has been steadily increasing over the years (Armstrong et al., 2023). Clinically, DFU repair progresses through four distinct phases: coagulation, inflammation, proliferation, and remodeling (Liang et al., 2021; Ramachandran et al., 2023). In advanced DM, the generation of advanced glycation end-products and mitophagy dysfunction contribute to pathological changes, such as an imbalanced inflammatory response, elevated OS, impaired VEC regeneration, and compromised fibroblast repair (Magalhaes-Novais et al., 2022; Chen et al., 2022a). During the inflammatory phase, it is beneficial to reduce inflammatory factors by upregulating basal mitophagy, which can mitigate inflammation. Onishi et al. (2021) demonstrated that dysfunction in mitophagy leads to the generation of damaged mitochondria, resulting in the release of mitochondrial ROS and DNA. This triggers excessive activation of the NLRP3 inflammasome, thereby increasing the creation of pro-inflammatory cytokines (PICs) like IL-1β/18. Additionally, mitophagy orchestrates mitochondrial metabolism, influencing the differentiation of immune cells. M2 macrophages, which rely on oxidative phosphorylation for their biosynthetic needs, are inhibited in their transformation under dysfunctional mitophagy, leading to a predominance of M1 macrophages. This shift enhances the inflammatory response (Xu et al., 2020; Peng et al., 2021). In the proliferative phase, moderately regulating mitophagy, often in response to local hypoxia at the wound site, promotes the repair process by alleviating apoptosis, facilitating collagen synthesis, and encouraging neovascularization. Xiang et al. (2022) reported that HG intervention brought about a notable reduction in the expression of mitophagy proteins (Beclin1, PINK1/Parkin, and LC3-II/I) in VECs. This resulted in mitochondrial dysfunction, increased endothelial cell apoptosis, and decreased cell migration and activity. During the remodeling phase, excessive autophagy can lead to mitochondrial loss and increased cellular energy consumption, which may exacerbate fibroblast apoptosis and hinder scar formation in diabetic skin ulcers (Liu et al., 2019a). Yinji Luo et al. found that (Luo et al., 2024) HG exposure reduced cell viability in DFU rats, with elevated ROS, SA-β-gal, p21, p62 proteins, but decreased LC3II/I levels. HG inhibited mitophagy by accelerating dermal fibroblast senescence via the inhibition of the PINK1/Parkin axis. However, the administration of ASC-EVs facilitated mitophagy via activating the PINK1/Parkin axis *in vivo*, thereby improving DFU pathology. Conclusively, mitophagy is intricately involved in various stages of DFU development. Therefore, regulating mitophagy could be a promising strategy for improving DFU outcomes.

### 3.5 Other categories

DM can lead to various ocular complications, including cataracts, glaucoma, and iridocyclitis. Mitophagy is critical in the degeneration of ocular tissues (Brooks et al., 2023). It has been demonstrated that a hyperglycemic environment can reduce ATP synthesis, decrease MMP, and alter mitochondrial autophagic flux in lens ECs (LECs), ultimately resulting in LEC apoptosis (Xu et al., 2018; Liu et al., 2020). Aldrich et al. (2017) observed that mitophagy was impaired in human corneal endothelial cells from DM patients. The mitochondria in these cells appeared swollen and contained dark inclusions, suggesting a disruption of mitophagy. Furthermore, Hu et al. (2019) reported that HG diminished the expression of Sirt3 in TKE2 mouse corneal ECs, which in turn reduced Parkin/PINK1-mediated mitochondrial autophagic flux. However, overexpression of Sirt3 activated the Parkin/PINK1 axis, enhancing mitophagy and promoting corneal epithelial wound healing. These findings highlight that modulation of mitophagy could serve as an effective therapeutic strategy to mitigate ocular complications in DM patients.

## 4 Natural small molecules intervene in chronic complications of DM by modulating autophagy

**FIGURE 3:**
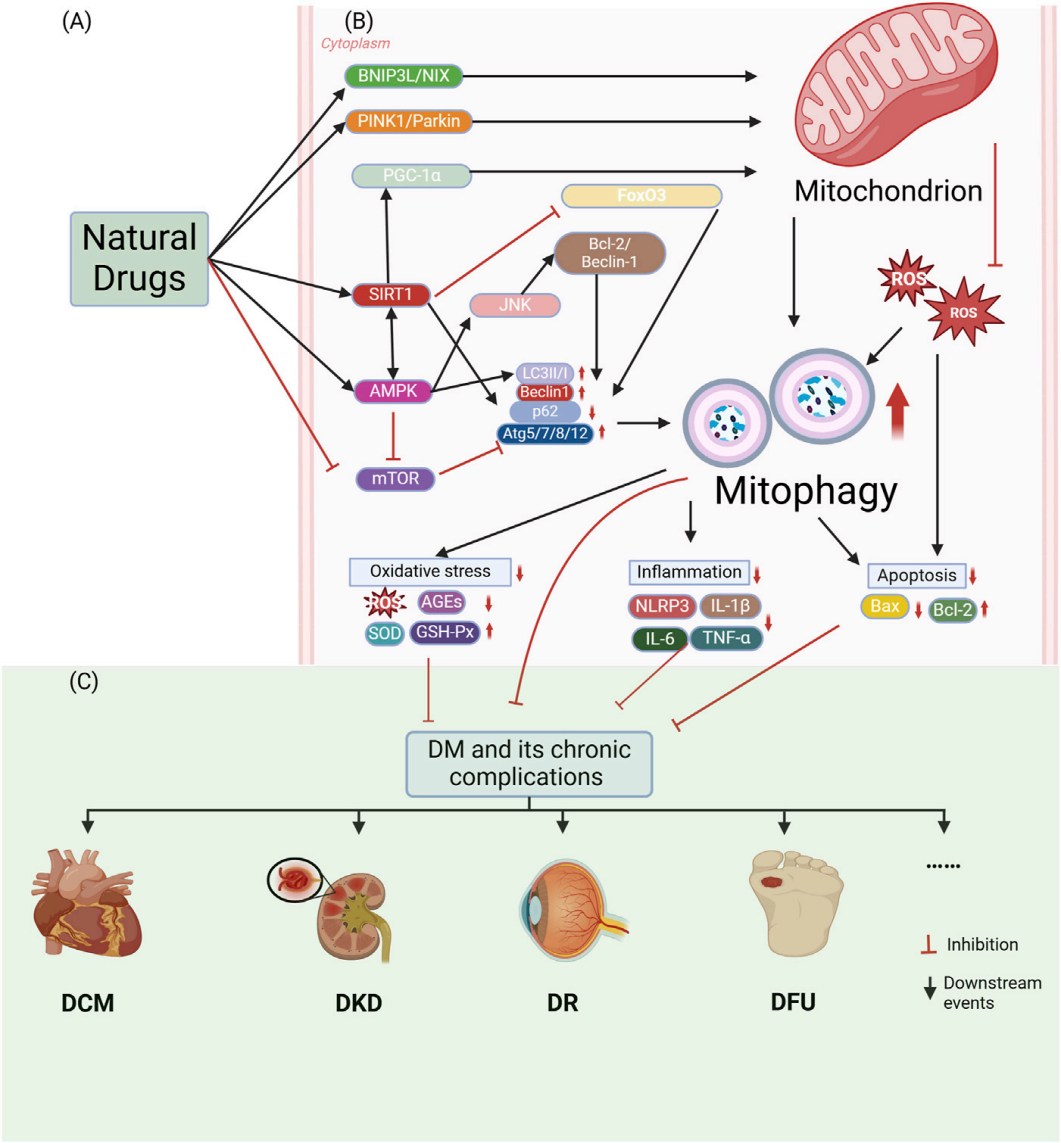
Natural small molecules modulate diabetes and chronic complications through activation of mitophagy. **(A)** Natural small molecules function as therapeutic interventions targeting mitochondrial homeostasis. **(B)** Molecular mechanisms of mitophagy involve: Core regulators: PINK1/Parkin signaling, BNIP3L/NIX-mediated mitochondrial recognition, LC3II/I lipidation, Beclin1-dependent autophagy initiation, and Atg5/7/8/12-p62 complexes; Pathogenic drivers: Oxidative stress (ROS/AGEs accumulation, SOD/GSH-Px depletion) and inflammatory activation (NLRP3 inflammasome, IL-1β/IL-6/TNF-α overproduction). **(C)** Pharmacological activation of mitophagy attenuates diabetic pathogenesis and chronic complications through mitochondrial quality restoration. The figure is drawn with biorender.com.

A growing body of research has demonstrated that natural small molecules can orchestrate mitophagy to effectively treat DM and its chronic complications. Below is an overview of how these natural compounds impact mitophagy in DM and its associated complications. Natural small molecules regulate diabetes and chronic complications by activating mitophagy are illustrated in Figure 3.

### 4.1 Metabolic damage in DM

Ginsenosides (Table 1), the primary bioactive constituents of *Panax ginseng* (Fan et al., 2023), are renowned for their antioxidant, anti-inflammatory, and anticancer attributes (Sarhene et al., 2021; Oh and Chun, 2022). These compounds have been shown to exert diverse beneficial effects, including the enhancement of insulin sensitivity, promotion of glucose uptake, and mitigation of OS through the reduction of ROS (Paik et al., 2023; Liu et al., 2019b; Zhou et al., 2019a). Additionally, ginsenosides have been reported to support lipid metabolism by lowering triglyceride (TG) and cholesterol levels, bolster mitochondrial function, and safeguard cardiovascular wellbeing by enhancing endothelial function and diminishing blood pressure (Sarhene et al., 2021; Xue et al., 2021; Hou et al., 2020). In a particular study, DM mice were administered ginsenosides (10 mg/kg) daily for 4 weeks. The findings revealed that ginsenosides facilitated the translocation of glucose transporter 4 to the plasma membrane in fatty acid-treated C2C12 cells, thereby enhancing glucose uptake and glycogen synthesis, in addition to improving mitochondrial quality. Notably, the DRP1/PINK1 axis was identified as a critical mediator in the ginsenoside-induced mitophagy process (Li et al., 2023a).

**TABLE 1:** Source, classification and structure of compounds for the treatment of diabetes mellitus.

No.	Natural drugs	Sources	Classifications	Genes regulated	Molecular function	Mode of action	Structures	The source of 3D molecular structure	References
1	Ginsenosides	*Panax ginseng*	Triterpenoid saponin	PINK1, DRP1, FUNDC1, BNIP3L/NIX	Promote mitophagy	Activation of the DRP1/PINK1 pathway, upregulates FUNDC1 and BNIP3L/NIX expression, facilitating damaged mitochondrial clearance and alleviating oxidative stress	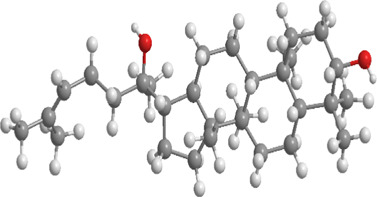	pubchem.ncbi.nlm.nih.gov/compound/3086007#section=3D-Conformer	Li et al. (2023a)
2	Silymarin	*Silybum marianum*	Flavonoid	PINK1, Parkin	Promote mitophagy	Activation of the PINK1/Parkin pathway promotes mitochondrial autophagy-mediated clearance of damaged organelles, inhibits ferroptosis, and reduces ROS levels	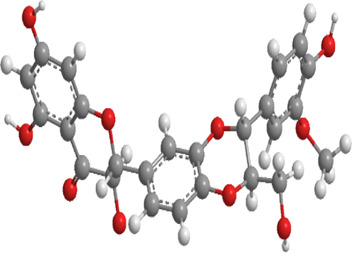	pubchem.ncbi.nlm.nih.gov/compound/5213	Du et al. (2023)
3	Punicalagin	Pomegranate skin polyphenols	Ellagitannin	PINK1, Parkin, BNIP3, LC3II, p62.	Promote mitophagy	Activation of the PINK1/Parkin pathway enhances BNIP3, LC3b, p62, MnSOD, and CAT expression, mitigating oxidative damage	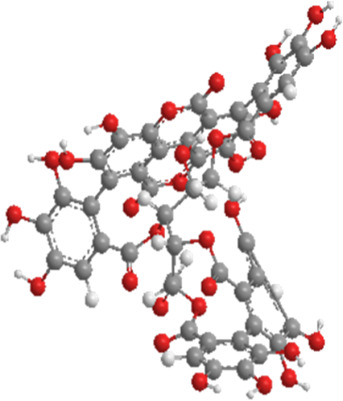	pubchem.ncbi.nlm.nih.gov/compound/16129719	Zhang et al. (2022c)
4	Mangiferin	Mango	Xanthone	PINK1, PRKN, LC3-II, p62, MFN1, MFN2, OPA1, DRP1, FIS1	Inhibit mitophagy	Inhibition of PINK1-Parkin-mediated mitophagy balances mitochondrial fusion/fission dynamics, stabilizes mitochondrial membrane potential (MMP), and suppresses ROS generation	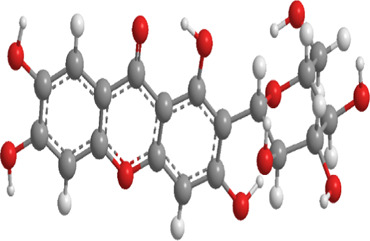	pubchem.ncbi.nlm.nih.gov/compound/5281647	Rahman and Kim (2020)
5	Berberine (medicine)	Chinese goldthread (Coptis chinensis), rhizome used in medicine	Alkaloid	PINK1, Parkin, LC3II/I, p62, Mfn1, Mfn2	Promote mitophagy	Activation of the PINK1/Parkin pathway reduces ROS production, maintains MMP and ATP synthesis, and enhances antioxidant capacity via Nrf2 signaling	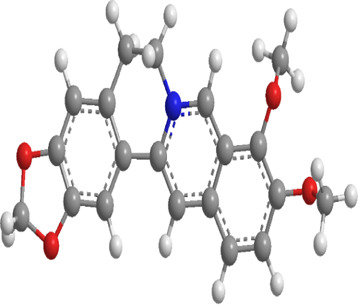	pubchem.ncbi.nlm.nih.gov/compound/2353#section=3D-Conformer	Li et al. (2022)
6	Melatonin	Pineal body	Amines	SIRT3, Parkin, LC3-II, p62, PGC-1α.	Promote mitophagy	activates mitophagy and alleviates oxidative stress	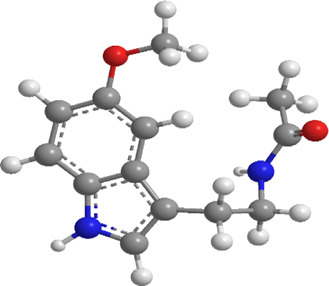	pubchem.ncbi.nlm.nih.gov/compound/896#section=3D-Conformer	Song et al. (2023)
7	Nobiletin	Orange, Lemon	Flavonoid	PINK1, Parkin, LC3-II, p62, ATG3.	Promote mitophagy	Activation of the PINK1/Parkin pathway sustains MMP and ATP production while suppressing NLRP3 inflammasome-driven inflammatory signaling	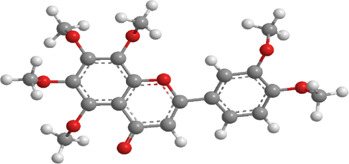	pubchem.ncbi.nlm.nih.gov/compound/72344#section=3D-Conformer	Yuan et al. (2022b)
8	Puerarin	Tuber of the kudzu vine (Pueraria lobata) used in Chinese medicine	Flavonoid	PINK1, Parkin、LC3, Beclin 1, p62	Promote mitophagy	Activation of the PINK1/Parkin pathway regulates Drp1 expression, maintains mitochondrial dynamics, optimizes mitophagic flux, and attenuates oxidative stress	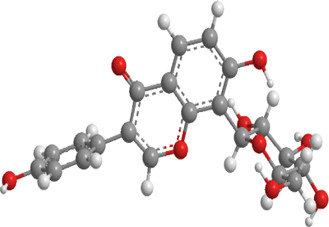	pubchem.ncbi.nlm.nih.gov/compound/5281807#section=3D-Conformer	Chen et al. (2018b)
9	Resveratrol	*Vitis Ampelopsis*	Polyphenol	BNIP3L, NIX, PINK1, Parkin.	Inhibit mitophagy	Inhibition of BNIP3L/NIX, PINK1, and Parkin expression	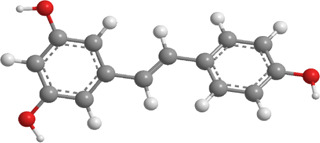	pubchem.ncbi.nlm.nih.gov/compound/445154#section=3D-Conformer	Wang et al. (2018a)
10	Cyanidin 3-glucoside chloride	Blueberries	Flavonoid	PINK1.PARKIN, LC3.	Promote mitophagy	Activation of the PINK1/Parkin pathway enhances clearance of damaged mitochondria and ameliorates oxidative stress	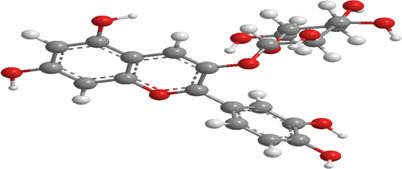	pubchem.ncbi.nlm.nih.gov/compound/197081#section=3D-Conformer	Ye et al. (2021)

Silymarin (Table 1), a flavonol glycoside derived from *Silybum marianum* (Bijak, 2017), possesses hepatoprotective, neuroprotective, anti-inflammatory, anticancer, and islet-protective properties. In a study using the rat pancreatic β-cell line INS-1, cells were exposed to 0.4 mM palmitic acid (PA) and 25 mM HG for 24 h, with silymarin administered 4 h prior to PA and HG treatment. The results showed increased levels of total iron, lipid ROS, MDA, and COX-2, alongside decreased levels of iron death inhibitory molecules such as GSH, GPX4, and FSP1. Moreover, PINK1/parkin-mediated mitophagy was repressed. The protective effect of silymarin against PA and HG-induced ferroptosis was shown to be dependent on mitophagy, as evidenced by silencing PINK1 expression using mitophagy agonists and inhibitors like urolithin A (UA) and cyclosporine A (CsA), as well as siRNA transfection (Du et al., 2023).

Punicalagin (PU) (Table 1), a hydrolyzable tannin derived from pomegranate rind polyphenols, is metabolized *in vivo* to ellagic acid and urolithin, and exerts antioxidant, anti-inflammatory, and lipid metabolism-regulating properties (Alami et al., 2024; Cerdá et al., 2003; Johanningsmeier and Harris, 2011). In animal studies, DM mice were treated with PU (20 mg/kg) via gavage once a day for 8 weeks. Cellular experiments with HepG2 cells exposed to HG for 48 h and treated with PU revealed significant reductions in FBG, fasting serum insulin, and the homeostasis model assessment of IR in DM mice. Additionally, serum and liver levels of markers such as ALT, AST, TC, TG, LDL-C, FFA, MDA, and total superoxide dismutase (T-SOD) were notably reduced. Liver steatosis and inflammation were also mitigated. Importantly, MMP was distinctly increased, and levels of PINK1, Parkin, BNIP3, LC3B, P62, MnSOD, and CAT were evidently elevated in liver tissue. The activities of MnSOD and CAT were also notably elevated in both serum and liver, correlating with the *in vitro* findings. These results suggest that mitophagy and antioxidant enzyme activities were elevated, offering protection against diabetic liver injury (Zhang et al., 2022c).

Mangiferin (MF) (Table 1), a flavonoid derived from mango, exhibits diverse biological activities, including anticancer, antidiabetic, and anti-obesity effects (Sim et al., 2019; Ramírez et al., 2017; Sekar et al., 2019; Du et al., 2018). In a study involving mouse C3H10T1/2 mesenchymal stem cells (MSCs), MF was incorporated into a lipid-inducing medium (MDI) for 2 to 4 consecutive days, followed by maturation culture. The results identified that MF treatment induced the expression of brown adipose markers such as UCP1, TG, PGC1α, PRDM16, and PPARγ, in addition to upregulating beige adipose markers (CD137, HSPB7, TBX1, and COX2) in both C3H10T1/2 MSCs and human adipose-derived MSCs (hADMSCs). MF modulated mitochondrial fusion dynamics by repressing PINK1-Parkin-mediated mitophagy, thus increasing mitochondrial DNA levels and improving mitochondrial homeostasis. Furthermore, there was an enhancement in mitochondrial respiration, demonstrated by elevated mitochondrial oxygen consumption and an elevation of oxidative phosphorylation (OXPHOS)-related proteins. Through chemical inhibition and knockdown assays, it was revealed that the β3-AR-dependent PKA-p38 MAPK-CREB signaling, mediated by mitophagy, is pivotal in MF-mediated brown fat formation (Rahman and Kim, 2020).

Berberine (BBR) (Table 1), an isoquinoline alkaloid primarily extracted from *Berberis vulgaris* (Zhu et al., 2019), is well recognized for its broad pharmacological effects, which include anti-inflammatory, antioxidant (Tian et al., 2019), and modulatory actions on glucose and lipid metabolism (Wu et al., 2016). In one experiment using the rodent pancreatic β-cell line INS-1, cells were pretreated with 5 µM of BBR for 1 h, followed by exposure to 0.5 mM PA for 24 h. The results indicated that BBR significantly improved cell viability under PA-induced stress, suppressed apoptosis, and enhanced insulin secretion. Additionally, BBR alleviated oxidative damage by reducing ROS production and boosting the activities of antioxidant enzymes. It was also observed that BBR restored ATP production and MMP, both of which were diminished upon PA treatment. Further analysis revealed that BBR induced mitophagy in PA-treated INS-1 cells, as evidenced by alterations in the expression levels of mitophagy-related proteins. Moreover, BBR promoted the nuclear translocation and DNA-binding activity of Nrf2, a key antioxidant transcription factor involved in mitophagy regulation. Interestingly, the protective effects of BBR on cell viability, apoptosis, and mitochondrial function were abolished by silencing PINK1 expression, underscoring the critical role of mitophagy in mediating its beneficial actions (Li et al., 2022).

Melatonin (Mel) (Table 1), a hormone synthesized by the pineal gland and secreted during the night (Reiter et al., 2009), plays essential roles in regulating the sleep-wake cycle, as well as possessing antioxidant and immunomodulatory properties. In a study containing T2DM rats, Mel (20 mg/kg) was administered daily for 1 week. Results showed that SIRT3 signaling and mitophagy were suppressed after diabetic lung I/R injury (LIRI). Mel treatment distinctly induced mitophagy and restored SIRT3 expression. This intervention improved lung function recovery, suppressed inflammation, reduced oxidative damage, decreased apoptosis, and preserved mitochondrial function, thus attenuating the progression of diabetic LIRI (Song et al., 2023).

Nobiletin (Table 1), a polymethoxyflavonoid derived from the peels of oranges and lemons, is known for its antihypertensive (Potue et al., 2019), anti-cancer (Shi et al., 2013), anti-inflammatory (Nohara et al., 2019; Namkoong et al., 2017), anti-obesity, and anti-aging properties. In animal studies, DM mice were orally administered nobiletin (100 or 150 mg/kg) once daily for 3 weeks. In cellular experiments, NIT-1 cells were pretreated with 10 or 12 µM nobiletin for 3 days. The results indicated that nobiletin evidently alleviated hyperglycemia in DM mice and effectively activated mitophagy in NIT-1 cells. It also suppressed inflammatory pathways and restored MMP that had been disrupted by glucotoxicity in the cells. The hypoglycemic potentials of nobiletin seemed to be mediated by the modulation of intestinal microbiota dysbiosis, activation of mitochondrial autophagic processes, repression of inflammatory vesicle expression, and restoration of pancreatic islet morphology in DM mice (Yuan et al., 2022b).

Puerarin (Table 1), an isoflavonoid predominantly found in the roots of *Pueraria lobata* (Willd.), has long been used both as a dietary supplement and a traditional remedy for managing diabetic conditions (Zhou et al., 2014; Zhang et al., 2013; Wong et al., 2011). Studies have demonstrated that *P. lobata* improves insulin signaling dysfunction in skeletal muscle, particularly in HFD/STZ-induced DM rats and palmitate-treated insulin-resistant myotubes (Chen et al., 2018a). In a controlled study, rat L6 skeletal muscle cells were pre-exposed to 0.3 mM puerarin for 24 h, followed by treatment with 0.75 mM palmitate for an additional 24 h. The results demonstrated that puerarin pretreatment significantly improved insulin sensitivity and alleviated palmitate-induced mitochondrial dysfunction in muscle cells. This was evidenced by enhanced complex I activity, elevated MMP, increased ATP production, and a reduction in ROS. Furthermore, puerarin upregulated the expression of mitochondrial biogenesis-related key genes, oxidative phosphorylation, and ROS detoxification. The compound also modulated mitochondrial dynamics, regulating both fusion and fission processes, and reversed palmitate-induced impairments in mitophagy via the PINK1/Parkin axis. Additionally, puerarin mitigated palmitate-induced inflammation by suppressing the TLR4/NF-κB axis (Chen et al., 2018b).

Resveratrol (RES) (Table 1), a naturally occurring polyphenolic antioxidant found in peanuts, pine, and grape skins (Wang et al., 2014; Alamdari et al., 2012; Russell et al., 2006; Wyke and Tisdale, 2006), has garnered significant interest due to its antioxidant properties and its ability to repress protein degradation, thereby mitigating muscle fiber atrophy in various *in vitro* systems. In recent years, RES has been recognized for its potential to counteract muscle wasting in conditions such as DM, cancer, and cachexia (Momken et al., 2011; Shadfar et al., 2011; Chen et al., 2011; Bennett et al., 2013; Joseph et al., 2013). In one experiment, diabetic mice were fed a diet containing 0.04% RES for a period of 8 weeks. The findings indicated that RES addition evidently reduced muscle atrophy and improved overall skeletal muscle function. This was achieved through the reduction of key markers such as ubiquitin and muscle ring finger protein-1 (MuRF-1), along with a decline in LC3-II and cleaved caspase-3 levels. Additionally, RES treatment promoted mitochondrial biogenesis and mitigated excessive mitophagy activation in the skeletal muscle. Notably, RES also protected muscle tissue from abnormal mitochondrial dynamics, including excessive fusion and fission in DM (Wang et al., 2018a).

Cyanidin 3-glucoside chloride (C3G) (Table 1), a typical anthocyanin, is considered one of the most significant flavonoids with potential health benefits across a range of diseases (Guo and Ling, 2015). Accumulating evidence has demonstrated that C3G exhibits antioxidant, anti-inflammatory, hepatoprotective, and anticancer properties, partly attributed to its free radical scavenging ability (Wang et al., 2019; Li et al., 2020a). In one study, DM mice were given an aqueous solution of C3G (150 mg/kg) once daily for 6 weeks. C3G was capable of activating mitophagy by elevating the expression of PINK1 and PARKIN, as evidenced by the accumulation of LC3 and a reduction in mitochondrial number. The autophagy inhibitor chloroquine (CQ) blocked C3G-induced mitophagy and inhibited the ability of C3G to reduce ROS production, suggesting that mitophagy is crucial in the alleviation of OS in islet cells by C3G (Ye et al., 2021).

### 4.2 Microvascular lesions

#### 4.2.1 DKD

Sulforaphane (SFN) (Table 2), an isothiocyanate derived from cruciferous vegetables, is a potent antioxidant with potential protective effects against DM-induced cellular damage (Gu et al., 2017). Previous studies have documented the nephroprotective properties of SFN in DKD (Li et al., 2020b). In one study, DM mice were treated with different doses of SFN (6.25 or 12.5 mg/kg) three times a week for 12 weeks. The results revealed that overexpression of PINK1 in Nrf2 conditional knockout (cKO) mice resulted in a reduction of the urinary albumin/creatinine ratio, blood urea nitrogen, and serum creatinine levels. Furthermore, PINK1 overexpression diminished p62 protein levels and attenuated mitochondrial damage, increasing the number of membrane-intact mitochondria and mitochondrial autophagic vesicles. SFN was shown to activate mitophagy in podocytes, restore urinary albumin levels, and prevent glomerular hypertrophy and excessive pedunculated fusion in DM mice. The nephroprotective effect of SFN was abolished in podocyte-specific Nrf2 cKO mice, suggesting that SFN mitigates DM-induced podocyte damage through the Nrf2/PINK1 axis. *In vitro*, SFN treatment elevated PINK1 expression and activated mitophagy in HG-treated podocytes. Additionally, SFN translocated Nrf2 to the nucleus, where it bound to the PINK1 promoter, enhancing PINK1 transcription. Conclusively, SFN attenuates podocyte damage in DKD by regulating the Nrf2/PINK1 axis and balancing mitophagy, thereby preserving mitochondrial homeostasis (Wang et al., 2023c).

**TABLE 2:** Source, classification and structure of compounds for the treatment of diabetic nephropathy.

No.	Natural drugs	Sources	Classifications	Genes regulated	Molecular function	Mode of action	Structures	The source of 3D molecular structure	References
1	Sulforaphane	Cruciferous vegetables	Isothiocyanate	Nrf2, PINK1, LC3, p62	Promote mitophagy	Activation of the Nrf2/PINK1 pathway alleviates oxidative stress	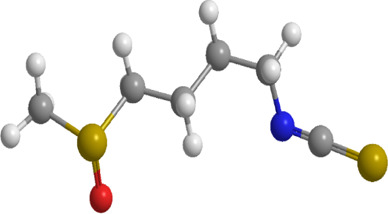	pubchem.ncbi.nlm.nih.gov/compound/5350#section=3D-Conformer	Wang et al. (2023c)
2	Astragaloside II	Milk vetch root (used in TCM)	Triterpenoid saponin	Nrf2, PINK1, Parkin, LC3, p62	Promote mitophagy	Upregulation of PINK1 and Parkin activates the Nrf2/PINK1 pathway, thereby alleviating oxidative stress	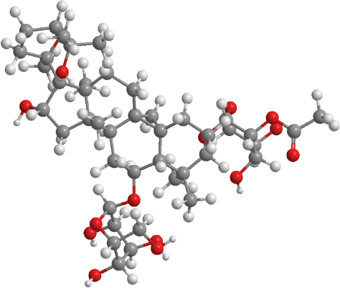	pubchem.ncbi.nlm.nih.gov/compound/13996693	Su et al. (2021)
3	Icariin	Epimedium, genus of herbaceous flowering plant, cultivated in the Far East as aphrodisiac	Flavonoid	Sesn2, Nrf2, HO-1, PINK1, Parkin	Promote mitophagy	Upregulation of Sesn2 expression promotes mitophagy through the PINK1/Parkin pathway and inhibits NLRP3 inflammasome activation	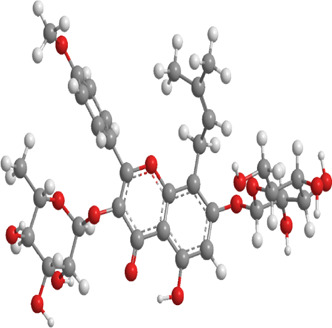	pubchem.ncbi.nlm.nih.gov/compound/5318997#section=3D-Conformer	Ding et al. (2022)
4	Diosgenin	Chinese yam (Dioscorea opposita)	Steroidal saponins.	Parkin, PINK1, DRP1, MFN2	Promote mitophagy	Downregulation of Bax, CytC, Apaf-1, caspase-9, p-PERK, p-EIF2α, IRE1, p-IRE1, XBP1s, ATF4, p-CHOP, and caspase-12	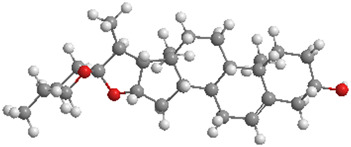	pubchem.ncbi.nlm.nih.gov/compound/99474#section=3D-Conformer	Zhong et al. (2022a)
5	Jujuboside A	Ziziphus spinosa	Triterpenoid saponins	Parkin, PINK1, LC3, AMPK, mTOR, CaMKK2	Promote mitophagy	Upregulation of SOD, CAT, and GPx, along with downregulation of NOX4, Bax, CytC, Apaf-1, and caspase-9, activates the CaMKK2/AMPK/p-mTOR and PINK1/Parkin pathways, thereby alleviating oxidative stress	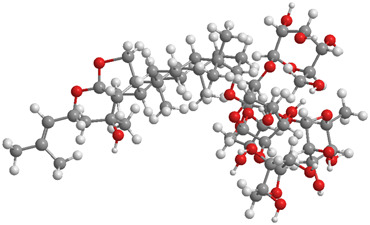	pubchem.ncbi.nlm.nih.gov/compound/51346169	Zhong et al. (2022b)
6	Isoorientin	Polygonum orientale	Flavonoid	PI3K, AKT, TSC2, mTOR, LC3, p62, TOM20, TIM23	Promote mitophagy	Inhibition of the PI3K-AKT-TSC2-mTOR pathway increases the LC3II/LC3-I ratio and reduces p62 expression, thereby enhancing mitophagy	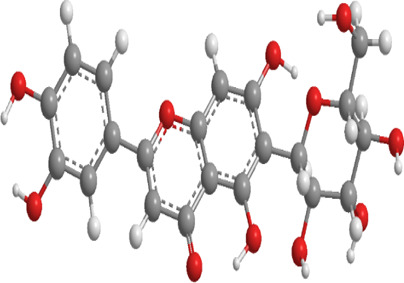	pubchem.ncbi.nlm.nih.gov/compound/114776	Kong et al. (2023)
7	Astragaloside IV	Milk vetch root (used in TCM)	Triterpenoid saponin	Drp-1, Fis-1, MFF, PINK1, Parkin	Inhibit mitophagy	Downregulation of PINK1 and Parkin and expression decreases the levels of Drp-1, Fis-1, and MFF, thereby reducing mitophagy	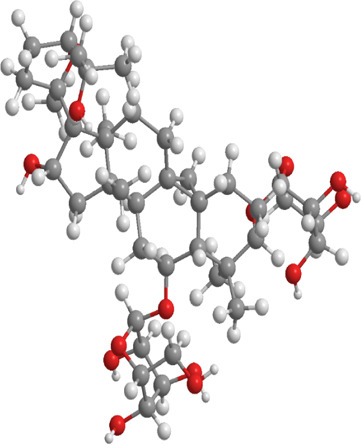	pubchem.ncbi.nlm.nih.gov/compound/13943297	Liu et al. (2017b)
8	Orientin	Fenugreek	Flavonoid	LC3, p62	Promote mitophagy	Regulate the LC3II/LC3-I ratio and reduce p62 expression	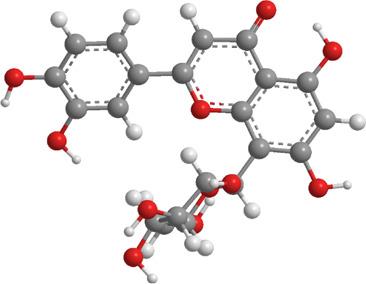	pubchem.ncbi.nlm.nih.gov/compound/5281675	Kong et al. (2020)

Astragaloside II (AS II) (Table 2), a bioactive compound derived from *Astragalus*, is well known for its anti-inflammatory and wound-healing capabilities, particularly in the context of inflammatory bowel disease (Qiao et al., 2019; Lee et al., 2017). In a study involving DM rats, AS II was administered daily at doses of 3.2 or 6.4 mg/kg over a period of 9 weeks. The results revealed that AS II treatment partially restored the expression of critical mitochondrial proteins involved in mitochondrial dynamics, such as Mfn2, Fis1, P62, and LC3. Furthermore, AS II brought about elevations in the expression of PINK1 and Parkin, which are vital for the modulation of mitophagy in these rats. The treatment also elevated Nrf2 expression and suppressed Keap1, thereby promoting resistance to OS. These findings highlight that AS II may help mitigate podocyte damage and mitochondrial dysfunction in DM rats, likely through the modulation of the Nrf2 and PINK1/Parkin signaling (Su et al., 2021).

Mel (Table 1), a hormone produced by the pineal gland, is central to regulating circadian rhythms and has critical roles in mitochondrial function (Reiter et al., 2018), energy metabolism (Cipolla‐Neto et al., 2014), lipid regulation (Jin et al., 2017), and reproduction (Olcese, 2020). Mitochondrial dysfunction (Zhou et al., 2019b), abnormal energy homeostasis (Bhargava and Schnellmann, 2017), and lipid metabolism (Herman-Edelstein et al., 2014) are intimately linked to DKD progression. Mel is capable of ameliorating hyperglycemia-induced steroidogenesis impairment in Leydig cells by activating the SIRT1 axis (Wang et al., 2022b). In a study on DM mice with kidney injury, Mel (0.2 mg/kg) was administered daily for 12 weeks. The results demonstrated that Mel treatment stimulated AMPK phosphorylation, which facilitated the translocation of PINK1 and Parkin to mitochondria, thus activating mitophagy. This process alleviated OS and reduced inflammatory responses. Notably, the nephroprotective effects of Mel were partially diminished when PINK1 was diminished or AMPK was inhibited, indicating that Mel’s protective action in DKD is, at least in part, mediated through the AMPK/PINK1/mitophagy axis (Tang et al., 2022).

Icariin (ICA) (Table 2), a flavonoid extracted from the traditional Chinese medicine (TCM) plant *Epimedium* (Qi et al., 2021a), has been shown to improve lipid metabolism disorders, reduce inflammation, enhance insulin sensitivity, and alleviate mitochondrial dysfunction (Wang et al., 2020; Qiao et al., 2018; Han et al., 2015). ICA also acts on glomerular plasma membrane cells via the TGF-β1/Smad2 axis, increasing gelatinase activity and promoting the degradation of excess extracellular matrix (ECM) (Li et al., 2013). In a study involving DM rats treated with different doses of ICA (20, 40, 80 mg/kg) once daily for 8 weeks, ICA evidently elevated the expression of LC3II, Sesn2, PINK1, and Parkin, while downregulating the inflammatory markers NLRP3, caspase-1 (both pro-caspase-1 and its active form, caspase-1 p10), and IL-1β in a dose-responsive fashion. Conclusively, ICA may attenuate the inflammatory response by inducing autophagy. ICA also activates Nrf2, suppresses NLRP3, and promotes Keap1 degradation through Sesn2-dependent mitophagy. When mitophagy signaling was blocked by Sesn2 siRNA, the results demonstrated that ICA-induced repression of NLRP3 and mitophagy was diminished, confirming the involvement of Sesn2 in this process (Ding et al., 2022).

Diosgenin (Table 2), a steroidal saponin (Mahmoudi et al., 2021), is recognized for its anticancer properties (Zheng et al., 2023a). Diosgenin exerts its therapeutic benefits in type II diabetic nephropathy by targeting CaMKK2 and enhancing autophagy, mitochondrial dynamics, and mitophagy (Zhong et al., 2023). In a study focusing on DM rats, dioscin (20 mg/kg) was administered daily for 8 weeks. The results demonstrated that dioscin mitigated mitochondrial and endoplasmic reticulum stress (ERS)-induced apoptosis by diminishing the expression of pro-apoptotic proteins, including Bax, CytC, Apaf-1, caspase-9/12, pPERK, p-EIF2α, IRE1, p-IRE1, XBP1s, ATF4, and p-CHOP. Additionally, dioscin improved mitochondrial quality and quantity by modulating key mitochondrial proteins like Parkin, PINK1, DRP1, p-DRP1, and MFN2 (Zhong et al., 2022a).

Jujuboside A (JuA) (Table 2), a triterpenoid saponin isolated from Jujube seeds, is renowned for its antioxidant, anti-inflammatory, and anti-apoptotic attributes (Zhong et al., 2022b). JuA has shown potential in mitigating HFD/STZ-induced diabetic nephropathy, by inhibiting OS, reducing apoptosis, and promoting autophagy. In a study with diabetic Sprague-Dawley rats, JuA (20 mg/kg) was administered once daily for 8 weeks. The findings revealed that JuA enhanced mitochondrial respiratory chain function by modulating the expression of respiratory chain complexes, reducing levels of superoxide anion and H_2_O_2_, and inducing the activities of antioxidant enzymes like SOD, CAT, and GPx, while decreasing NOX4 expression. Furthermore, JuA diminished mitochondrial apoptotic proteins, including Bax, CytC, Apaf-1, and caspase-9. Additionally, JuA promoted mitophagy through the CaMKK2/AMPK/p-mTOR and PINK1/Parkin pathways (Zhong et al., 2022b).

Isoorientin (ISO) (Table2),a flavonoid also known as 3′,4′,5,7-tetrahydroxyflavone-6-D-glucopyranoside or lignan 6-C-glucoside, is a constituent of *fenugreek* (Ziqubu et al., 2020). ISO has been found to help prevent metabolic complications such as hyperglycemia, hyperlipidemia, and IR ((Malik et al., 2019; Yuan et al., 2016). Its therapeutic effects are attributed to its antioxidant and anti-inflammatory properties (Alonso-Castro et al., 2012). In a study involving DM mice treated with varying doses of ISO (10, 20, or 40 mg/kg) once daily for 2 months, ISO was shown to promote autophagy in podocytes and protect them from HG-induced injury. Under HG conditions, ISO improved the autophagic clearance of damaged mitochondria. ISO also reversed hyperphosphorylation of TSC2 S939 and stimulated autophagy by inhibiting the PI3K-AKT-TSC2-mTOR axis. Additionally, ISO is validated to bind to the SH2 structural domain of PI3Kp85β, which is critical for its recruitment (Kong et al., 2023).

BBR (Table 1), an alkaloid extracted from the stem bark, roots, and rhizomes of plants in the Berberidaceae family (Neag et al., 2018), exhibits diverse pharmacological effects, including antidiabetic (Kapoor et al., 2014), antimicrobial (Xia et al., 2022), antihyperlipidemic (Wu et al., 2022b), anticancer (Saxena et al., 2018), antihypertensive (Fatehi-Hassanabad et al., 2005), antidepressant (Sun et al., 2014), and neuroprotective effects. In an experimental study, NRK-52E cells were exposed to varying concentrations of D-glucose (5.5, 30, 40 mM) for 24 h, followed by a co-treatment with 20 µM BBR for an additional 24 h. The results indicated that BBR provided protective effects in hyperglycemic cells by ensuring mitochondrial structure and function. Co-treatment with SRT-1720, a SIRT1 activator, enhanced autophagy, decreased apoptosis, elevated the expression of downstream proteins such as FoxO3a and Bnip3, and alleviated mitochondrial dysfunction. Conversely, the use of nicotinamide, a SIRT1 inhibitor, reversed these beneficial effects. Additionally, a GFP reporter gene assay showed that BBR intervention increased the transcriptional activity of FoxO, which was linked to elevated Bnip3 expression. Knockdown of FoxO3a resulted in impaired autophagy and increased apoptosis. Pre-treatment with N-acetyl-L-cysteine demonstrated that ROS were involved in the HG-induced cellular toxicity in NRK-52E cells. Moreover, co-treatment with BBR led to changes in the expression of key proteins related to autophagy and mitophagy, including LC3B, ATGs, Beclin1, Sirt1, Bnip3, FoxO3a, and Parkin. Transmission electron microscopy confirmed the enhancement of mitophagy following BBR treatment (Saxena et al., 2024).

Astragaloside IV (AS-IV) (Table 2), a lanolinoloidal tetracyclic triterpenoid saponin derived from *Astragalus membranaceus*, has diverse pharmacological activities, including anti-inflammatory, antioxidative, anti-apoptotic, and anti-fibrotic properties (Li et al., 2017a). AS-IV is capable of improving DKD in animal models through its anti-inflammatory mechanisms (Gui et al., 2013), inhibition of ERS (Wang et al., 2015), and protection of podocytes (Chen et al., 2014; Gui et al., 2012). In a study, DM mice were administered AS-IV (1 g/kg) for 12 weeks. The results showed that AS-IV distinctly limited urinary albumin excretion and urinary N-acetyl-β-D-glucosaminidase, while ameliorating renal pathology. Moreover, AS-IV administration was linked reduced expression of mitochondrial fission regulators, including Drp-1, Fis-1, and MFF in DM mice. PINK1/Parkin-mediated mitophagy was found to be abnormally activated in the diabetic group. AS-IV administration notably decreased the expression of Drp-1, Fis-1, and MFF and diminished PINK1/Parkin-mediated mitophagy. This suggests that AS-IV may delay the progression of DKD by modulating mitochondrial dynamics and autophagy in DM mice (Liu et al., 2017b).

Trigonella foenum-graecum (fenugreek), an important treatment for DKD (Jiang et al., 2018), contains *Orientin* (Table 2), a C-glycosylated flavonoid, which is the primary bioactive component. Orientin has demonstrated antidiabetic, antioxidant, and autophagy-inducing effects (Zhong et al., 2019; Li et al., 2019b; Lawal et al., 2019; Kasangana et al., 2019). In one study, MPC-5 cells were incubated with 30 mM glucose and 120 µM orientin. It was found that Orientin restored cell proliferation in HG-induced conditions by reducing apoptosis. Orientin also protected mitochondrial membrane integrity in HG-treated cells, likely through its role in autophagy. Furthermore, Orientin distinctly enhanced cellular resistance to apoptosis and promoted autophagy by regulating mitochondria in podocytes. The protective effects of Orientin were reversed by 3-MA, an autophagy inhibitor, confirming the involvement of autophagy in its protective action (Kong et al., 2020).

#### 4.2.2 DR

Allicin (Alc) (Table 3), a natural compound found in garlic, is recognized for its antioxidant and anti-inflammatory properties, making it a promising therapeutic agent for DR. In one study, DM rats were treated with Alc (16 mg/kg) for 28 days. Alc treatment effectively ameliorated histopathological changes and metabolic abnormalities associated with T2DM. It diminished focal death-associated proteins, elevated mitophagy-related proteins, reduced PIC levels, and attenuated OS. The antioxidant and anti-inflammatory effects of Alc were, in part, mediated by the activation of the mitophagy axis through the PINK1/Parkin signaling cascade. In conclusion, Alc mitigates DR in rats by activating PINK1/Parkin-mediated mitophagy and inhibiting OS-induced inflammation, specifically through the NOD-like receptor family pyrin structural domains (Xu and Yu, 2024).

**TABLE 3:** Source, classification and structure of compounds for the treatment of diabetic retinopathy.

No.	Natural drugs	Sources	Classifications	Genes regulated	Molecular function	Mode of action	Structures	The source of 3D molecular structure	References
1	Allicin	Allium sativum	Organic sulfur	PINK1, Parkin,	Promote mitophagy	Activation of the PINK1/Parkin signaling pathway promotes mitophagy, thereby reducing oxidative stress and inflammatory responses	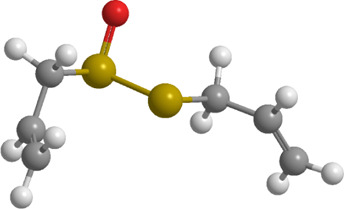	pubchem.ncbi.nlm.nih.gov/compound/65036	Xu and Yu (2024)
2	Notoginsenoside R1	Panax notoginseng	Triterpenoid saponin	PINK1, Parkin, LC3, p62, SQSTM1	Promote mitophagy	Upregulation of PINK1, Parkin, and LC3-II/LC3-I, along with downregulation of p62 and SQSTM1	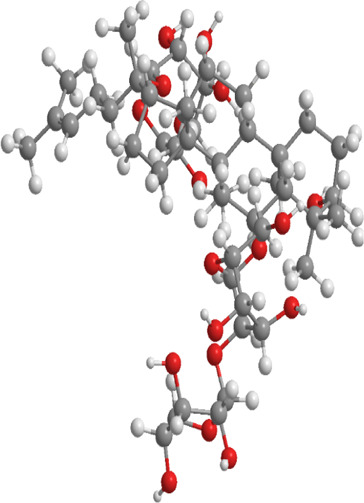	pubchem.ncbi.nlm.nih.gov/compound/441934	Zhou et al. (2019c)

Notoginsenoside R1 (NGR1) (Table 3), a novel saponin extracted from *P. ginseng*, has demonstrated therapeutic effects, including the treatment of diabetic encephalopathy and the improvement of microcirculatory disorders. In one study, DM mice were treated with NGR1 (30 mg/kg) daily for 12 weeks. The findings revealed that NGR1 effectively reduced apoptosis, suppressed VEGF expression, and elevated the levels of pigment epithelium-derived factor. Additionally, NGR1 treatment alleviated OS and inflammation in rat retinal Müller cells (rMC-1) exposed to HG and in the retinas of DM mice. Moreover, NGR1 administration increased the expression of PINK1 and Parkin in both HG-treated rMC-1 cells and DM mouse retinas. This was accompanied by a higher LC3-II/I ratio and reduced levels of p62/SQSTM1. Furthermore, NGR1 promoted the co-localization of GFP-LC3 and MitoTracker in rMC-1 cells. Notably, silencing PINK1 abolished the protective potentials of NGR1, suggesting that NGR1 mitigates DR by enhancing mitophagy via a PINK1-dependent mechanism (Zhou et al., 2019c).

Mel (Table 1) has been demonstrated to be effective in the management of DR. Mel helps preserve the integrity of the blood-retinal barrier via diminishing hypoxia-inducible factors (HIF-1α, HIF-1β), VEGF, and its receptor genes. Additionally, Mel downregulates genes associated with mitochondrial fission (e.g., DRP1, hFis1, MIEF2, MFF) and mitophagy (such as PINK1, BNip3, and NIX), while promoting the expression of genes associated with mitochondrial biogenesis, including PGC-1α, NRF2, and PPAR-γ, thus supporting mitochondrial homeostasis. In an *in vitro* model of diabetic macular edema induced by hyperglycemia and hypoxia, Mel also mitigated blood-retinal barrier dysfunction and mitochondrial damage, demonstrating its protective effects (Doğanlar et al., 2021).

Several natural small molecules also show promise in treating DR. Cannabis derivatives play significant neuroprotective and neuroregenerative roles by reducing neurotoxicity, inflammation, and blood-retinal barrier disruption in diabetic animals, likely through inhibition of MAPK signaling. Cannabidiol may alleviate vascular leakage in DR, with its effects potentially linked to antioxidant, anti-inflammatory properties, and modulation of mitophagy (Kokona et al., 2016; Ramirez et al., 2022). Finally, RSV has been shown to promote mitophagy via SIRT1 activation, increase AMPK activity, inhibit NF-κB to control inflammation, and reduce ROS production, thus exerting protective effects on retinal ganglion cells (Guo et al., 2022).

#### 4.2.3 DCM

RES (Table 1) is a biologically active natural polyphenolic compound known for its antioxidant properties, which can attenuate OS in DCM (Su et al., 2022). In one study, DM mice were injected with RES (50 mg/kg) once daily for 7 days. The results validated that RES elevated mitochondrial Parkin and diminished p62 in myocardial tissues of DM mice, suggesting that RES promotes mitophagy in diabetic myocardium. Additionally, myocardial p53 expression was elevated in DM mice, but RES diminished p53 expression and its binding to Parkin. This reduction in p53 binding to Parkin was inferred to enhance mitophagy, thereby alleviating diabetic myocardial injury (Wu et al., 2020).

Mel is a potent antioxidant with established benefits across various diseases (Fu et al., 2020). In one study, diabetic myocardial infarction mice were treated with Mel (50 mg/kg) once daily for 4 weeks after myocardial infarction surgery. The results revealed that Mel diminished intracellular levels of Bax, caspase-3, and p62, while upregulating Bcl-2, LC3-II/I, and Parkin. Furthermore, Mel distinctly increased intracellular ATP content (Jiao et al., 2022). These findings suggest that Mel can effectively reduce mitochondrial dysfunction and inhibit cardiomyocyte damage after myocardial infarction by enhancing mitophagy.

Fucoxanthin (FX) (Table 4), a carotenoid derived from marine sources, is known for its potent antioxidant properties and has been studied for its therapeutic significance in DCM. In an experimental study, DM rats were administered varying doses of FX (200 mg/kg) daily for 12 weeks. The results demonstrated that FX activated Nrf2 signaling, leading to a reduction in ROS levels. Additionally, FX enhanced Bnip3/Nix pathways, which contributed to improved mitochondrial function and a decrease in both mitochondrial and intracellular ROS, effectively reversing HG-induced hypertrophy in H9c2 cells. However, the application of CQ, an autophagy inhibitor, negated the anti-hypertrophic effects of FX, resulting in compromised mitochondrial function and elevated ROS levels. Furthermore, FX treatment reduced the accumulation of key fibrosis markers, including TGF-β1, fibronectin (FN), and α-smooth muscle actin (α-SMA), thus attenuating myocardial fibrosis in STZ-induced DM rats. FX also promoted mitophagy through the elevation of Bnip3/Nix and enhanced Nrf2-mediated signaling to mitigate OS, which contributed to the inhibition of HG-induced hypertrophy in H9c2 cells (Zheng et al., 2022).

**TABLE 4:** Source, classification and structure of compounds for the treatment of atherosclerotic cardiovascular diseases.

No.	Natural drugs	Sources	Classifications	Genes regulated	Molecular function	Mode of action	Structures	The source of 3D molecular structure	References
1	Fucoxanthin	Brown algae	Carotenoids	Bnip3, Nix, LC3, Nrf2	Promote mitophagy	Upregulation of Bnip3/Nix and activation of the Nrf2 signaling pathway enhance mitophagy and alleviate oxidative stress	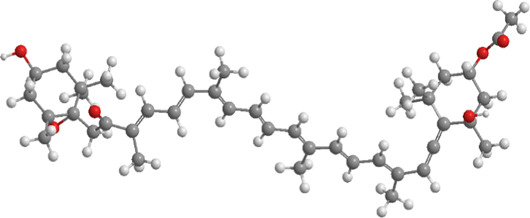	pubchem.ncbi.nlm.nih.gov/compound/5281239	Zheng et al. (2022)
2	Salvianolic acid B	Salvia miltiorrhiza	Phenolic acid	p-DRP1, FIS1, ROCK1	Inhibit mitophagy	Inhibition of the ROCK1 pathway reduces the expression of p-DRP1 and FIS1, decreases mtROS and mtDNA release, and inhibits mitophag	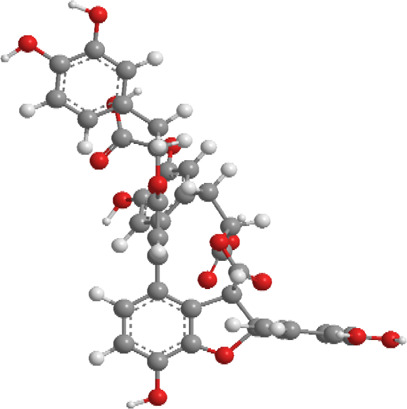	pubchem.ncbi.nlm.nih.gov/compound/6451084	Ko et al. (2020)

### 4.3 ASCVDs

Scutellarin, a natural compound, exhibits excellent antioxidant (Wang et al., 2016; Mo et al., 2018), anti-inflammatory (Zhang et al., 2017), vasodilator (Koon et al., 2014), antidiabetic activity (Yang et al., 2017), and vasoprotective (Du et al., 2015) properties. In one study, human umbilical vein endothelial cells (HUVECs) were subjected to HG treatment to induce VEC injury *in vitro*, followed by Scutellarin treatment (30 μM) for 48 h. The results demonstrated that Scutellarin notably improved cell viability in HG-exposed HUVECs. Additionally, Scutellarin diminished apoptosis-related proteins like Bcl-2, Bax, and cytochrome C (Cyt.c), thereby inhibiting cell death through a mitochondrial-dependent mechanism. Furthermore, Scutellarin alleviated OS by enhancing SOD activity, upregulating SOD2, and reversing the loss of MMP. Scutellarin also promoted autophagic flux, as evidenced by elevations in the levels of LC3-II, Beclin 1, and autophagy-related gene 5 (Atg5), while decreasing the levels of Sequestosome1/P62 in HG-treated HUVECs. Moreover, Scutellarin elevated key proteins involved in mitophagy, including PINK1, Parkin, and Mitofusin 2, indicating an enhancement of MQC. Silencing PINK1 diminished the beneficial effects of Scutellarin, specifically attenuating the HG-induced Parkin under-expression, ROS overproduction, and the elevated levels of p62, Cyt. c, and cleaved caspase-3. Molecular docking studies further revealed a strong binding interaction between baicalin and PINK1 proteins, suggesting a direct involvement in the modulation of mitochondrial function (Xi et al., 2021).

Mel (Table 1) has also been shown to ameliorate cardiomyocyte damage. In one study, DM mice were administered Mel (20 mg/kg) once daily for 4 weeks. In cellular experiments, primary cardiomyocytes were treated with Mel (100 μmol/L) for 4 h, followed by HG incubation for 48 h. The results revealed that Mel increased the clearance of dysfunctional mitochondria, promoted LC3-II expression, and enhanced the co-localization of mitochondria and lysosomes in HG-challenged cardiomyocytes. Mel also increased the number of typical autophagosomes phagocytosing mitochondria in the hearts of DM mice. These findings pinpoint that Mel promotes mitophagy. Knockdown of Parkin abolished the beneficial potentials of Mel in cardiac mitochondrial morphology and bioenergetic disturbances, eliminating the significance of Mel on remodeling in DCM hearts. Moreover, Mel inhibited the phosphorylation of mammalian sterile 20-like kinase 1 (Mst1), thereby facilitating Parkin-mediated mitophagy, which contributes to MQC (Wang et al., 2018b).

BBR (Table 1) has also demonstrated beneficial effects on cardiomyocytes. In one study, HG-induced injury was modeled in the H9C2 cardiomyocyte cell line. BBR was pretreated at 100 nM for 30 min, followed by the addition of 50 mM D-glucose for 24 h. The results demonstrated that BBR corrected the imbalance between mitochondrial fusion and fission, significantly alleviating hypertrophy in the H9C2 cells and improving mitochondrial function. BBR further promoted mitochondrial biogenesis and cleared damaged mitochondria through mitophagy. This effect was mediated via the activation of the AMPK axis, which restored autophagic flux in HG-induced cardiomyocyte injury (Hang et al., 2018).

Salvianolic acid B (Sal B) (Table 4), from the roots and rhizomes of *Salvia divinorum* (family Labiatae), possesses antioxidant, anti-inflammatory, and antithrombotic properties. In one study, DM mice were administered Sal B (50 mg/kg) once daily for 14 days. It was found that Sal B reduced inflammatory cell infiltration in tissues and facilitated angiogenesis. In both diabetic tissues and HG-induced human microvascular endothelial cells, Sal B improved apoptosis and enhanced mitophagy. Inhibition of Parkin impaired cell migration, promoted apoptosis, and inhibited mitophagy in human microvascular endothelial cells (Zhang et al., 2025). Moreover, Sal B prevented oxLDL-induced endothelial dysfunction under HG conditions by diminishing ROCK1-mediated mitochondrial dynamics and apoptosis (Ko et al., 2020).

### 4.4 Neurological complications

Baicalin, a naturally occurring flavonoid from *Scutellaria baicalensis* Georgi, is recognized for its neuroprotective properties. In an experimental study, DM rats were administered baicalin (100 mg/kg) daily for 1 week, while PC12 cells were cultured under HG conditions and treated with baicalin for 24 h. The results demonstrated that baicalin administration effectively reduced blood glucose levels, mitigated neurological impairments, and decreased infarct size. *In vitro*, under OGD/R conditions, ROS production and mitochondrial dysfunction were notably elevated in HG-treated PC12 cells. However, baicalin treatment suppressed the expression of dynamin-related protein 1 (Drp-1), which is involved in mitochondrial fission, while enhancing mitochondrial fusion by promoting the production of MFN2. Additionally, baicalin increased Drp-1 Ser637 phosphorylation, contributing to improved MMP by reducing ROS production (Li et al., 2017b).

Piceatannol (PCN) (Table 5), found in foods such as peanuts, mountain grapes, and lingonberries, is a natural analog of RES. It exhibits antioxidant, anticancer, anti-inflammatory, and neuroprotective properties (Jia et al., 2020; Setoguchi et al., 2014; Suh et al., 2018; Uchida-Maruki et al., 2015). In one study, DM rats were treated with various doses of PCN (10 or 20 mg/kg) once daily for 2 weeks. The results showed that PCN exposure restored mitochondrial function in HG-induced N2A cells by reducing ROS production, restoring mitochondrial superoxide levels, and improving MMP. Additionally, PCN exposure promoted neurite growth and induced mitochondrial biogenesis through PGC-1α activation, which was mediated by enhanced SIRT1 activation. SIRT1 activation also enhanced Nrf2-mediated antioxidant signaling, thereby counteracting mitochondrial dysfunction and reduced antioxidant activity in DM rats and HG-treated N2A cells (Khan et al., 2023).

**TABLE 5:** Source, classification and structure of compounds for the treatment of diabetic neuropathy.

No.	Natural drugs	Sources	Classifications	Genes regulated	Molecular function	Mode of action	Structures	The source of 3D molecular structure	References
1	Piceatannol	Grapes, blueberries	Polyphenol	PINK1, Parkin, PARL, PGAM5	Promote mitophagy	Activation of SIRT1 regulates the PINK1-Parkin pathway and the PARL-PGAM5 axis, thereby alleviating oxidative stress	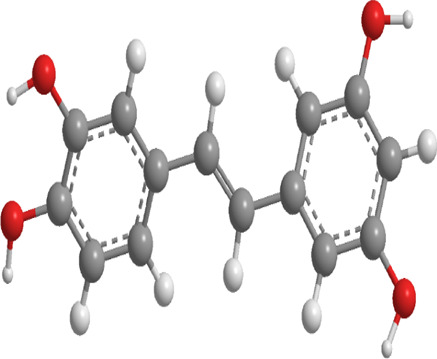	pubchem.ncbi.nlm.nih.gov/compound/667639	Khan et al. (2023)
2	Isoliquiritigenin	Glycyrrhiza glabra Mongolian glycyrrhiza	Flavonoid	SIRT1, AMPK, PGC-1α, mTOR	Promote mitophagy	Activation of SIRT1 and AMPK, along with inhibition of mTOR, promotes mitophagy and reduces oxidative stress	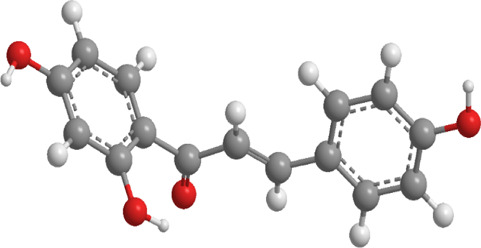	pubchem.ncbi.nlm.nih.gov/compound/638278#section =3D-Conformer	Yerra et al. (2017)

AS-IV (Table 2) also plays a significant role in diabetic neuropathy (DN). In a cellular experiment, Xuewang cells were treated with 50 μmol/L AS-IV for 72 h. The results showed that MMP was elevated in both the AS-IV and N-acetylcysteine (NAC) treatment groups, with MtMP evidently higher in the AS-IV group relative to the HG group (*p* < 0.01). AS-IV exhibited a protective effect on mitochondrial function, significantly increasing MtMP in Schwann cells (SCs) under HG conditions. Furthermore, the expression of autophagy-related proteins such as LC3, PINK1, and Parkin was diminished in the AS-IV group, suggesting that AS-IV inhibits the over-activation of autophagy in SCs. This reduction in autophagy-related proteins was linked to a notable decrease in ROS levels and improvements in mitochondrial morphology and membrane potential. These findings unfold that AS-IV protects mitochondrial integrity in SCs by reducing OS and autophagy activation in a HG environment (Wei et al., 2022).

Isoliquiritigenin (ILQ) (Table 5), a licorice-derived compound, is recognized for its potent antioxidant, anti-inflammatory, and anticancer capabilities (Kwon et al., 2009; Watanabe et al., 2016). It has also been investigated for its potential antidiabetic effects and other therapeutic benefits (Gaur et al., 2014). In a recent study, DM rats were treated daily with different doses of ILQ (10 or 20 mg/kg) for 2 weeks. The findings revealed that ILQ treatment notably activated SIRT1, which in turn enhanced mitophagy. Additionally, ILQ administration increased the NAD+/NADH ratio in the peripheral sciatic nerve. Functional and behavioral analyses showed that ILQ improved nerve conduction, restored blood flow to the nerves, and alleviated nociceptive hypersensitivity and abnormalities in the DM rats. In cultured N2A cells, ILQ was found to mitigate HG-induced ROS generation and mitochondrial membrane depolarization. Overall, ILQ’s activation of SIRT1 mimicked the effects of caloric restriction, promoting PGC-1α-mediated mitochondrial biogenesis, FOXO3a-mediated stress resilience, and AMPK-mediated autophagy, which collectively counteracted several pathophysiological features of experimental DN (Yerra et al., 2017).

Mel (Table 1) has also been implicated in DN. In an experiment using DMEM incubated for 24 h, HG intervention notably elevated PINK1 and LC3B expression. In addition, HG exposure reduced the levels of cytochrome c oxidase subunit 4 and decreased Mitotracker™ fluorescence intensity. Silencing PINK1 expression resulted in a marked accumulation of mitochondrial ROS, disruption of MMP, and an increase in the expression of cleaved caspase-3/9. Furthermore, the number of cells positive for the membrane-bound protein V was elevated. Notably, silencing PINK1 abolished the regulatory effects of Mel on mitochondrial ROS production, as well as its ability to activate cleaved caspase-3/9, and to increase the number of V-positive cells (Onphachanh et al., 2017).

### 4.5 DFUs and other categories

Cinnamaldehyde (Table 6), the principal bioactive compound in cinnamon, has long been recognized for its anti-inflammatory and wound-healing properties in TCM. Cinnamaldehyde exerts its beneficial effects by activating the PINK1/Parkin axis, which enhances mitophagy. This pathway activation leads to a decrease in PICs, such as IL-6 and TNF-α, while simultaneously increasing the expression of VEGF and collagen. These changes not only help in mitigating inflammation but also support angiogenesis and collagen production, ultimately accelerating wound healing in DM rats (Hong et al., 2023). RES (Table 1), a well-known natural antioxidant, has also demonstrated notable potential in treating diabetic ocular complications. Studies indicate that RES is effective in reducing ROS production in LECs exposed to HG conditions (Chen et al., 2022b). Moreover, RES improves mitophagy and decreases apoptosis in these cells, highlighting its therapeutic potential in managing diabetic eye diseases.

**TABLE 6:** Source, classification and structure of compounds for the treatment of diabetic foot ulcers.

No.	Natural drugs	Sources	Classifications	Genes regulated	Molecular function	Mode of action	Structures	The source of 3D molecular structure	References
1	Cinnamaldehyde	Cinnamomum cassia	Aldehyde	PINK1, Parkin, LC3Ⅱ	Promote mitophagy	Upregulation of the expression of PINK1, Parkin, and LC3-II	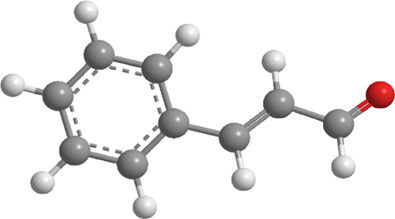	pubchem.ncbi.nlm.nih.gov/compound/637511	Hong et al. (2023)

In summary, Natural small molecules have shown potential in treating DM and its complications by regulating mitophagy. Current mechanistic studies have predominantly focused on the Pink1/Parkin-dependent pathway and the mitophagy receptor-dependent pathways (e.g., FUNDC1, BNIP3, NIX) to modulate mitochondrial dynamics in mammalian cells. The primary mechanism of action appears to involve alleviating OS, which in turn orchestrates mitophagy.

## 5 Clinical trials and patents

Natural small molecules demonstrate significant therapeutic potential for DM and its complications through modulation of mitophagy. However, clinical translation of these discoveries remains challenging. This Section summarizes the natural small molecules discussed in this review that have progressed to human clinical trials and analyzes key barriers to clinical validation, and examines the current status of related patents.

### 5.1 Natural small molecule clinical trials and challenges

Several natural small molecules have advanced to human clinical trials, demonstrating therapeutic potential for the management of DM and its associated complications. Clinical Research Summary of Mitophagy - Modulated Natural Small Molecules in Diabetes Mellitus are illustrated in Table 7.

**TABLE 7:** Mitophagy-modulated natural small molecules in diabetes mellitus: Clinical research summary.

Natural small molecule	Drug dose	Clinical trial results	References
Silymarin	140 mg 3 times daily for 45 days	Improvement in antioxidant markers (SOD, GPX and TAC) and reduction in hs-CRP levels in diabetic patients	Ebrahimpour et al. (2015)
Punicalagin	17.42 mg twice daily for 56 days	Improved inflammatory status and biomarker indicators of oxidative stress in diabetic patients	Grabež et al. (2022)
Berberine	500 mg 3 times daily for 84 days	Berberine reduced FPG and 2h-OGTT to levels below prediabetes diagnostic thresholds.	Panigrahi et al. (2023)
	500 mg 3 times daily for 91 days	Improved glycemic control, lipid metabolism and insulin sensitivity in diabetic patients.	Yin et al. (2008)
	600 mg 2 times daily for 84 days	Improved glycemic control and lipid metabolism in diabetic patients	Wang et al. (2022c)
Melatonin	3 mg daily for 1 weeks	Melatonin increased glycemic variability in individuals with T2DM	Martorina and Tavares (2023)
	6 mg daily for 8 weeks	Consumption of melatonin supplement may be effective in controlling arterial pressure including SBP, MAP, and PP and anthropometric indices (as predictors of obesity) in T2DM patients	Bazyar et al. (2021)
	10 mg daily for 12 weeks	Improvements in glycemic control, mental health, inflammatory markers, and oxidative stress in diabetic patients	Ostadmohammadi et al. (2020)
Resveratrol	200 mg once daily for 24 weeks.	Reduced insulin resistance and significant improvement in chronic inflammation, oxidative stress and associated microRNA expression in diabetic patients	Mahjabeen et al. (2022)
	500 mg daily for 6 months	Improvement of inflammation, renal function, glycemic parameters, insulin resistance, and nutrient sensing systems in elderly patients with type 2 diabetes	Ma and Zhang (2022)
Cannabidiol	The initial dose of 2.5 mg/kg/d was increased by 2.5–5.0 mg/kg every other day until the target dose of 20 mg/kg/d was reached. The duration was 4 weeks	Provides symptomatic relief and improves tolerance of fluid nutritional intake in patients with diabetic gastroparesis	Zheng et al. (2023b)
	100 mg twice daily for 13 weeks	Decreasing resistin and increasing glucose-dependent insulinotropic peptide in patients with type 2 diabetes mellitus.	Jadoon et al. (2016)

Although natural small molecules exhibit considerable therapeutic promise in preclinical research, their clinical translation faces five principal challenges: (1) Limited bioavailability due to inadequate absorption, non-targeted delivery mechanisms, off-target effects, and inherent toxicity (2) Significant pharmacokinetic variability affecting metabolic stability and therapeutic consistency (3) Insufficiently powered clinical trials with restricted longitudinal evaluation of safety profiles (4) Non-standardized extraction methodologies and formulation protocols leading to batch-to-batch variability (5) Absence of unified quality control standards compromising dosage reproducibility.

### 5.2 Patent status

The patent research, from the year 2023–2025, in the discipline of targeting the modulation of the mitophagy pathway using natural small molecules for the treatment of diabetes and its chronic complications, with the primary objective of either treating the condition or diminishing the disease progression, was conducted using the World Intellectual Property Organization’s official website. Patent Landscape Analysis of Mitophagy - Modulating natural small molecules for DM and Chronic Comorbidities is illustrated in Table 8.

**TABLE 8:** Patent Landscape Analysis of Mitophagy-Modulating natural small molecules for DM and Chronic Comorbidities.

Patent number	Applicants	Publication date	Patent name
US20250002490	Shenzhen Hightide Biopharmaceutical	02.January.2025	Berberine Salts, Ursodeoxycholic Salts and Combinations, Methods of Preparation and Application Thereof (Li, 2025)
US20230295151	Shenzhen Hightide Biopharmaceutical	21.September.2023	Pharmaceutical Composition Comprising Berberine and Ursodeoxycholic Acid for the Treatment of Various Diseases or Disorders (Li, 2023)
CN117695352	Beijing Chunfeng Pharmaceutical	15.March.2024	Traditional Chinese Medicine Preparation for Preventing Diabetes and Preparation Method Thereof (Yang, 2024)
AU2023200123	Shenzhen Hightide Biopharmaceutical	09.February.2023	Composition, and Application and Pharmaceutical Preparation Thereof (Liu, 2023)
WO2025024299	Arizona Board of Regents on Behalf of Arizona State University	30.January.2025	Resveratrol and Quercetin-Loaded Nanoparticles and Methods of Use Thereof (Wang et al., 2025)
IN202341049727	Chennupati Venkata Suresh, Ketan Girish Bhutkar, Pavan Kumar Padarthi, Heera Battu, Vivek Verma, Dhananjay Aoudumbar Chavan, Jagdish Chandra Nagar, Pravesh Kumar Sharma, Manish Dev Indoria, Manish Jaimini, Venkappa S Mantur, Badmanaban R, Chandan Komalkumar	01.September.2023	Pharmaceutical Composition for Managing Diabetes with Berberine and Gymnema Sylvestre Extracts (Chennupati et al., 2023)
WO2024141073	Shenzhen Hightide Biopharmaceutical	04.July.2024	Pharmaceutical Combinations and Compositions, and Methods of Use Thereof (Liu et al., 2024)
AU2023200826	Nanjing Nutrabuilding Bio-Tech Co.	09.March.2023	Administration of Berberine Metabolites (Harding et al., 2023)
US20240033313	Forte Capital	01.February.2024	Composition for Inhibiting Diabetes Mellitus and Method of Manufacturing the Same (Chen et al., 2024)
US20240139231	William H. Cross, III	02.May.2024	Metformin Compositions and Methods for Treatment of Diabetes (Cross, 2024)
IN202341015947	Velmurugan C	24.March.2023	Evaluation of Bergia Capensis on Hepatic Cholestasis Induced by Metformin Treated Diabetic Rats (Velmurugan et al., 2023)
RU0002822875	—	15.July.2024	Method for Preparing Microcapsules with Langerhans Islets and Microcapsule According to Disclosed Method (Casjm:yjlpca et al., 2024)

The therapeutic potential of natural small molecule in DM and its complications has garnered significant scientific interest, evidenced by exponentially increasing patent filings. Current intellectual property developments focus on three strategic domains: 1) Advanced drug delivery systems including nanoformulations and controlled-release matrices to enhance bioavailability (Uppal et al., 2018); 2) Synergistic combination therapies with conventional pharmacotherapeutics or nutraceutical adjuvants; 3) Novel therapeutic applications targeting diabetic complications such as cardiovascular pathologies, nephropathies, and neuropathies. Despite this surge in patent activity, translational implementation remains suboptimal, constrained by formulation challenges, market viability barriers, and intellectual property protection complexities.

In summary, mitophagy-modulating natural small molecules represent emerging therapeutic candidates for diabetes pathophysiology and its progressive sequelae. While select molecules have progressed to early-phase clinical evaluation, their therapeutic validation necessitates overcoming pharmacological barriers including suboptimal pharmacokinetic profiles, interindividual metabolic variability, and inadequately powered phase III trials.

## 6 Nanotechnology and natural small molecules

### 6.1 Nanotechnology, new opportunities for natural small molecules

**FIGURE 4:**
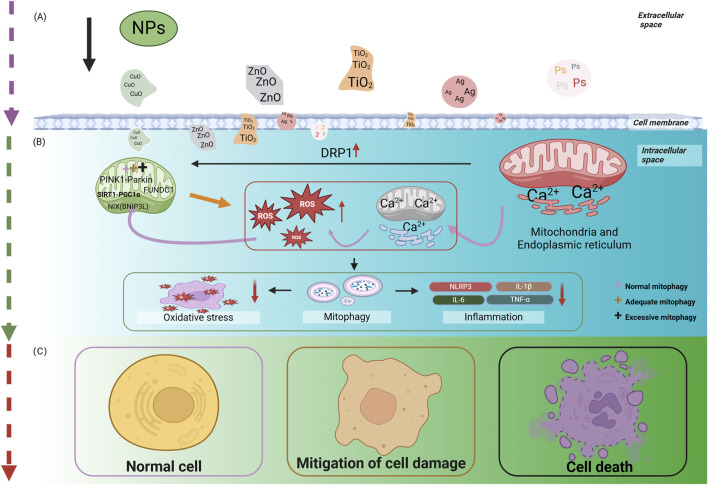
Illustrates the mechanistic basis of nanoparticle-mediated mitophagy regulation. **(A)** NPs penetrate the cell membrane. **(B)** NPs modulate the outcomes of mitophagy. **(C)** NPs regulate the outcomes of cells through mitophagy. PS-NPs, polystyrene nanoparticles; AgNPs, silver nanoparticles; TiO_2_NPs, titanium dioxide nanoparticles; CuONPs, copper oxide nanoparticles; DRP1, dynamin - related protein 1; NPs, nanoparticles; ROS, reactive oxygen species; ZnONPs, zinc oxide nanoparticles. The figure is drawn with biorender.com.

Natural small molecules exhibit unique therapeutic advantages, yet face pharmacological challenges including low bioavailability and poor targeting specificity. The integration of nanotechnology has revolutionized their delivery, particularly for mitophagy-inducing agents such as resveratrol, silymarin, and berberine (Habtemariam, 2020; Kumar et al., 2015; Tillhon et al., 2012; Patel et al., 2011; Ochi et al., 2016; Elgizawy et al., 2021). Despite their mitophagy modulation potential, clinical translation of these molecules remains constrained by inherent stability limitations and nonspecific biodistribution. Nanocarrier systems including liposomes, polymeric micelles, and mesoporous silica nanoparticles have emerged as viable solutions: MSN-based formulations enhance resveratrol solubility and controlled release (Juère et al., 2017), while liposomal encapsulation improves biocompatibility and enables pharmacokinetic optimization through compositional modifications (Lian and Ho, 2001; Handa et al., 2021; Cui et al., 2022). The mechanistic basis of nanoparticle - mediated mitophagy regulation is illustrated in Figure 4. Natural small molecules regulating mitophagy by nanotechnology is illustrated in Table 9.

**TABLE 9:** Natural small molecules regulating mitophagy by nanotechnology.

Nanocarriers	Materials	Major Natural Drugs	Advantages	References
Nanostructured lipid carriers	Hexadecyl palmitate; squalene; soybean phosphatidylcholine	Silibinin	Slower silibinin release; enhanced therapeutic efficiency and a reduction of adverse responses	Pan et al. (2016)
Liposomes	mPEG2000-DSPE; Dipalmitoylphosphatidylcholine; cholesterol	Silibinin	Increment of co-entrapment; suitable EE, stability, controlled release of drugs; lower IC50 on the HepG2 cancer cell line	Ochi et al. (2016)
Nanostructured lipid carriers	Soy lecithin; glyceryl tridecanoate; glyceryl tripalmitate; vitamin E acetate; Kolliphor HS15	Resveratrol	Showing stability in artificial gastric and/or intestinal fluids; significant anticancer activity against Hep-G2, human HCT-116, lymphoblastic leukemia cells (1,301), and human MCF-7 cell lines; significant apoptotic properties; potent *in vitro* antioxidant activity	Elgizawy et al. (2021)
Ethosomes	HSPC: DSPE-PEG2000; cholesterol	Cinnamaldehyde	Improved formulation stability and percutaneous drug absorption; increase interstitial cells of Cajal; excellent deformability; improved efficacy against UC	Zhang et al. (2020)
Mesoporous silica nanoparticles	Pluronic F127; CTAB; TWEEN20; MSNs	Resveratrol	Solubility, drug release, and transport enhancement of resveratrol; enhanced anti-inflammatory activity	Juère et al. (2017)
Mesoporous silica nanoparticles	Lipid-coated MSN@p (NIPAM-co-MA)	BBR	Improved efficacy and biocompatibility of the drug pair, desirable drug profiles at the low pH and higher temperature of the tumor microenvironment; excellent synergistic therapy effects *in vitro* and *in vivo*; lower systemic toxicity	Feng et al. (2019)
Nanocarriers based on active ingredients from TCMs	Polyethylene glycol-polylactic acid-co-glycolic acid	Baicalin	Significantly enhanced neuroprotective effects; improved brain targeting; sustained drug release; good biocompatibility; reduced systemic toxicity	Li et al. (2022)
Nanocarriers based on active ingredients from TCMs	BBR	BBR	Better inhibitory effect on multidrug-resistant S.qureus and stronger ability for biofilm removal; nonhemolytic with little toxicity *in vitro* and *in vivo*	Huang et al. (2020)

Diabetic heart disease exemplifies the critical role of mitochondrial dysfunction in disease pathogenesis, where impaired mitophagy exacerbates cardiac deterioration (Zorov et al., 2014; Mu et al., 2020) Natural small molecules demonstrate therapeutic potential by restoring mitophagy in cardiomyocytes. Specifically, resveratrol attenuates myocardial injury in diabetic murine models through p53/Parkin axis modulation, enhancing mitophagic flux (Wu et al., 2020). Nevertheless, the clinical utility of these molecules remains constrained by pharmacokinetic limitations. Nanocarrier-mediated delivery systems overcome these barriers by enabling cardiac-targeted RES, transport while stimuli-responsive nanoplatforms (e.g., pH- or ROS-activated systems) achieve spatiotemporal drug release, optimizing therapeutic outcomes (Uppal et al., 2018; Wu et al., 2022c; Qiu et al., 2023; Nag et al., 2024).

### 6.2 Challenges and limitations

Mitophagy research confronts three principal methodological and translational challenges. First, current detection modalities—including LC3-II/p62 immunoblotting and transmission electron microscopy—lack real-time monitoring capacity for autophagic flux dynamics and cannot distinguish mitophagy from general macroautophagy. Second, clinical translation barriers persist due to suboptimal bioavailability of natural small molecules, absence of validated biomarkers, and interpatient heterogeneity. Third, mechanistic knowledge gaps hinder targeted therapy development, particularly regarding crosstalk between mitophagy and parallel cellular processes (e.g., apoptosis-inflammation networks).

Emerging strategies address these limitations through (1) Nanodelivery platforms (liposomes, polymeric nanoparticles) enhancing molecular stability and tissue specificity (2) Prodrug engineering via strategic chemical modifications to optimize pharmacokinetics (3) Rational combination regimens integrating mitophagy modulators with adjuvants like antioxidants to potentiate therapeutic synergism. Complementary approaches employing CRISPR/Cas9-based gene editing and multiomics profiling are elucidating fundamental mitophagy mechanisms to inform clinical development.

## 7 Conclusion and outlook

In recent years, mitophagy has gained significant attention as a critical process in the development of various diseases. Much of the focus has been on understanding the Pink1/Parkin- and receptor-dependent pathways, such as those involving BNIP3, NIX, and FUNDC1, which play essential roles in modulating mitophagy. Growing evidence supports the idea that modulating mitophagy through natural small molecules could offer promising therapeutic modalities for addressing DM and its associated complications. Nevertheless, establishing the precise modulation thresholds of mitophagy for therapeutic intervention in diabetes mellitus and its chronic complications persists as a critical unresolved challenge in translational endocrinology. Despite these advancements, several critical questions remain unresolved regarding the mechanisms of mitophagy and the modulation of mitochondrial homeostasis (1) While current methodologies for assessing mitochondrial autophagic flux—including TOM20 degradation assays, mito-QC fluorescent probes, and mt-Keima imaging—provide discrete measurements, the field critically lacks a unified platform capable of real-time dynamic monitoring with integrated quantification. Current protocols necessitate multimodal verification through complementary techniques (e.g., immunoblotting, flow cytometry, electron microscopy), yet exhibit model-dependent variability in sensitivity and specificity across experimental systems, significantly complicating mechanistic investigations and pharmacological development (2) Most studies on the regulation of mitophagy by natural small molecules have been conducted in animal and cellular models, with limited multicenter clinical research. Future studies should involve larger sample sizes and multicenter trials to validate these findings in humans (3) The specific mechanisms linking mitophagy to DM remain unclear. The signaling pathways and molecular targets involved in autophagy are complex, and there is a need for targeted, systematic investigation into the autophagy-related pathways in DM. (4) Poor bioavailability and stability of natural compounds. Lack of targeted delivery mechanisms, Potential off-target effects, and toxicity concerns. Moving forward, expanding the research scope to explore the molecular mechanisms of mitophagy in greater depth is critical. The development of small molecule drugs targeting mitophagy regulation remains a key challenge. Future investigations should prioritize four strategic directions: First, leveraging artificial intelligence and machine learning to accelerate nanocarrier optimization and bioactive compound discovery. AI-powered molecular docking platforms enable high-precision prediction of natural small molecule interactions with mitophagy-related proteins (e.g., PINK1/Parkin), streamlining lead compound identification. Second, integrative approaches combining CRISPR-Cas9 screening, multi-omics profiling, and high-content histological analysis will elucidate mitophagy regulatory networks, facilitating novel target discovery. Third, advanced pharmaceutical engineering strategies—including prodrug design and stimuli-responsive nanodelivery systems—require development to overcome pharmacokinetic limitations of natural compounds. Fourth, synergistic therapeutic platforms merging nanotechnology-enhanced natural molecules with conventional agents hold promise for amplified clinical efficacy through multimodal mechanisms. Addressing these questions is essential for advancing our understanding of mitophagy-related diseases, identifying new therapeutic targets, and facilitating the clinical translation of novel drugs. These endeavors will not only improve the management of DM and its associated complications but will also offer valuable perspectives for future fundamental research in this domain.

## Glossary

DM: Diabetes mellitus
MMP: Mitochondrial membrane potential
MQC: Mitochondrial quality control
ROS: Reactive oxygen species
T1DM: Type 1 diabetes mellitus
GDM: Gestational diabetes mellitus
IR: Insulin resistance
OS: Oxidative stress
DKD: Diabetic kidney disease
EC: Epithelial cell
RTI: Renal tubular injury
DR: Diabetic retinopathy
VEC: Vascular endothelial cell
HG: High-glucose
HFD: High-fat diet
STZ: Streptozotocin
VEGF: Vascular endothelial growth factor
DCM: Diabetic cardiomyopathy
ASCVD: Atherosclerotic cardiovascular disease
H2S: Hydrogen sulfide
RAECs: Rat aortic endothelial cells
CNS: Central nervous system
DKA: Diabetic ketoacidosis
AD: Alzheimer’s disease
I/R: Ischemia/reperfusion
DPN: Diabetic peripheral neuropathy
HL: Hyperbaric lidocaine
DFU: Diabetic foot ulcer
PICs: Pro-inflammatory cytokines
LECs: Lens ECs
PA: Palmitic acid
UA: Urolithin A
CsA: Cyclosporine A
T-SOD: Total superoxide dismutase
MF: Mangiferin
MSCs: Mesenchymal stem cells
MDI: Medium
hADMSCs: Human adipose-derived MSCs
OXPHOS: Oxidative phosphorylation
BBR: Berberine
Mel: Melatonin
LIRI: lung I/R injury
RES: Resveratrol
MuRF-1: Muscle ring finger protein-1
C3G: Cyanidin 3-glucoside chloride
CQ: Chloroquine
SFN: Sulforaphane
cKO: Conditional knockout
AS II: Astragaloside II
ICA: Icariin
TCM: Traditional Chinese medicine
ECM: Extracellular matrix
ERS: endoplasmic reticulum stress
JuA: Jujuboside A
ISO: Isoorientin
AS-IV: Astragaloside IV
Alc: Allicin
NGR1: Notoginsenoside R1
rMC-1: rat retinal Müller cells
FX: Fucoxanthin
FN: Fibronectin
α-SMA: α-smooth muscle actin
HUVECs: Human umbilical vein endothelial cells
Cyt.c: Cytochrome C
Atg5: Autophagy-related gene 5
Mst1: Mammalian sterile 20-like kinase 1
Sal B: Salvia divinorum B
PCN: Piceatannol
DN: Diabetic neuropathy
NAC: N-acetylcysteine
SCs: Schwann cells
